# Effect of Cold-Sintering Parameters on Structure, Density, and Topology of Fe–Cu Nanocomposites

**DOI:** 10.3390/ma13030541

**Published:** 2020-01-23

**Authors:** Alexey Tsukanov, Dmitriy Ivonin, Irena Gotman, Elazar Y. Gutmanas, Eugene Grachev, Aleksandr Pervikov, Marat Lerner

**Affiliations:** 1 Center for Computational and Data-Intensive Science and Engineering (CDISE), Skolkovo Institute of Science and Technology (Skoltech), 30, bld. 1, Bolshoy Boulevard, 121205 Moscow, Russia; 2 Faculty of Physics, Lomonosov Moscow State University, GSP-1, 1-2 Leninskie Gory, 119991 Moscow, Russia; ivonin.dmitriy@physics.msu.ru (D.I.); grachevea@gmail.com (E.G.); 3 Department of Mechanical Engineering, ORT Braude College, Karmiel 2161002, Israel; irenag@braude.ac.il; 4 Department of Materials Science and Engineering, Technion-Israel Institute of Technology, Haifa 32000, Israel; gutmanas@technion.ac.il; 5 Institute of Strength Physics and Materials Science of SB RAS, 2/4, pr. Akademicheskii, 634055 Tomsk, Russia; pervikov@list.ru (A.P.); lerner@ispms.tsc.ru (M.L.)

**Keywords:** Fe–Cu nanocomposite, internal structure, bimetallic nanoparticles, cold sintering, high pressure, computer-aided design, Minkowski functionals, property prediction, computer modeling

## Abstract

The design of advanced nanostructured materials with predetermined physical properties requires knowledge of the relationship between these properties and the internal structure of the material at the nanoscale, as well as the dependence of the internal structure on the production (synthesis) parameters. This work is the first report of computer-aided analysis of high pressure consolidation (cold sintering) of bimetallic nanoparticles of two immiscible (Fe and Cu) metals using the embedded atom method (EAM). A detailed study of the effect of cold sintering parameters on the internal structure and properties of bulk Fe–Cu nanocomposites was conducted within the limitations of the numerical model. The variation of estimated density and bulk porosity as a function of Fe-to-Cu ratio and consolidation pressure was found in good agreement with the experimental data. For the first time, topological analysis using Minkowski functionals was applied to characterize the internal structure of a bimetallic nanocomposite. The dependence of topological invariants on input processing parameters was described for various components and structural phases. The model presented allows formalizing the relationship between the internal structure and properties of the studied nanocomposites. Based on the obtained topological invariants and Hadwiger’s theorem we propose a new tool for computer-aided design of bimetallic Fe–Cu nanocomposites.

## 1. Introduction

Composite engineering is a powerful approach to the design of materials with the required set of physicochemical properties. A composite material is a multi-component system that can exhibit enhanced performance compared to its individual constituents. However, the conventional powder metallurgy processing of stable multi-component systems is not always possible, as it is, for example, in the case of bimetallic nanocomposites of two immiscible metals or metals with limited mutual solubility [1].

Bimetallic Fe–Cu systems are a promising basis for creating materials that combine good mechanical strength, wear resistance, and corrosion resistance with high thermal and electrical conductivities [2]. Such materials can be used in a wide range of applications, from friction materials and high voltage sliding contacts to devices for magnetoelectronics and spintronics [3,4,5,6]. The creation of Fe nanostructures on Cu substrates is also of great theoretical and practical interest [7,8,9].

The physicochemical properties of bimetallic Fe–Cu composites depend on Fe-to-Cu ratio and internal structure of the material. In case of a nanostructured composite (a multi-component system with characteristic phase dimensions of about 1–100 nm), property prediction and computer-aided design become much more complex and require good understanding of the internal-structure properties relationship at the nanoscale. And the internal structure, in its turn, is determined by the processing method and the input parameters.

Cold sintering—consolidation of powders in a gradient of high pressure at ambient temperature (or temperatures significantly lower than the melting temperature, T << T_melt_)—is an attractive method for the preparation of bimetallic nanocomposites. In cold sintering, densities close to the theoretical can be achieved as a result of plastic deformation of powder particles and formation of strong interatomic bonds at the oxide-free interparticle interfaces [10,11]. Due to the low processing temperature, the nanoscale structure of starting nanopowders is retained in the final dense material [12].

The most important properties of a cold sintered product are relative density (expressed as a percentage of the consolidation density to the theoretical density, TD) and porosity. The density-consolidation pressure relationship is, therefore, a key characteristic of the cold-sintering process. Both fully dense and non-dense (porous) bimetallic structures are of interest for practical applications [13,14]. Fabrication of porous copper from a bimetallic Fe–Cu alloy was reported in [15].

The atomic-level structure of a bimetallic composite can be studied using atomistic molecular dynamics (MD) simulations. MD simulation of consolidation and densification of metallic nanopowders was reported by several researchers. The sintering behavior at T ≤ 900 K of many randomly oriented same-size copper nanoparticles was studied in [16], consolidation of an Al/SiC composite in a shock wave was simulated in [17]. Consolidation of a monodisperse Al nanopowder was simulated in [18], alloying in a single core-shell Ti–Al bimetallic nanoparticle was modeled by MD simulation in [19]. The use of numerical simulations for the investigation of metallic nanoparticles coalescence is reviewed in [20]. Despite numerous numerical studies in this direction, the problem of bimetallic nanocomposite formation from a bimetallic nanopowder has not been addressed yet.

The thrust of the present work is, therefore, to perform atomistic MD simulations of the cold-sintering processing of bimetallic Fe–Cu nanocomposites with the goal to establish the relationship between the properties of the bulk product (density, internal structure, i.e., atomic structure and the topology) and the input powder and cold-sintering parameters (elemental Fe–Cu powder composition and consolidation pressure). Previously, we reported the modeling of bimetallic Fe–Cu nanoparticles formation by the electrical explosion of wires (EEW) [21].

One of the approaches to the description of multi-component and multi-phase systems with complex structures is based on the analysis of their topological characteristics. This can be done, for example, using topological invariants, i.e., Minkowski functionals [22]. Minkowski functionals provide a convenient tool to characterize the structure and morphology of solid media [23] and are extensively used to model geological rocks [24,25]. If applied to materials science, Minkowski functionals can open new possibilities for establishing structure–properties relationships of materials and coatings. In [26], for example, topological invariants were used for the analysis of surface features in oxide films formed on anodization of Ni_3_Al intermetallic. In [27], Minkowski functionals were applied to structure characterization of thin tungsten carbide films. Minkowski functionals were also used to describe mechanical behavior and deformation of materials [28,29].

That is why we propose to evaluate four Minkowski functionals for the numerically simulated (synthetic) samples. The four functionals are specific volume, specific area, surface curvature, and the Euler characteristic calculated for the spatial distribution of both the elements and their phases. We would also like to obtain the dependence of these functionals on the cold-sintering parameters: consolidation pressure and elemental nanopowder composition. According to Hadwiger’s theorem [30], any additive, rotation, and translation invariant physical property can be represented as a linear combination of Minkowski functionals. Specifically, in our case Minkowski functionals and their dependence on the cold-sintering parameters can be used for estimating the properties and formalizing the structure–property relationship of Fe–Cu nanocomposites.

Adopting FAIR data principles [31], we propose to create a database of model cold-sintered Fe–Cu nanocomposites and their properties as well as a library of the corresponding Minkowski functionals.

## 2. Materials and Methods

### 2.1. Bimetallic Nanoparticles Powder Model

We have chosen bimetallic Fe–Cu nanoparticles reported in [21] as a basis for nanopowder models. The particles were obtained using a molecular dynamics simulation of nanoparticle formation by the collision and merging of monometallic nanoparticles in the course of the electrical explosion of wires. All the selected nanoparticles—three different sizes and three different component ratios—are Janus or quasi-Janus (having a copper shell). The nanoparticle types and parameters are described in Table 1.

To be able to adjust the component ratio for the initial model powder in a wider range, we added two large monometallic monocrystalline nanoparticles of pure iron Fe(100%) with body-centered cubic lattice (bcc) with a constant a = 2.855 Å (Table 1, row F), and pure copper Cu(100%) with face-centered cubic lattice (fcc), a = 3.615 Å (Table 1, row G), to the initial set of nanoparticles. It is worth mentioning that nanoparticles of pure metals may be found in bimetallic nanopowders as well, depending on the fabrication technology. A description of the initial set of nanoparticles used in the formation of model nanopowders is provided in Table 1.

We used different combinations of the above model nanoparticles to build seven nanopowder models where the content of iron varied between 21 and 80 at.%. Each nanopowder model contained 16 nanoparticles. The initial positions of nanoparticle centers in the simulation box were specified as if they were in nodes of a large bcc lattice with a constant of 180 Å. A view of the Fe–Cu 50/50 model in two projections is provided in Figure S1. A description of the composition and other parameters of model nanopowders, including an evaluation of (maximum) theoretical density (TD), is provided in Table 2 (and the supplementary data is provided in Table S1). In the calculations of TD, the following densities of pure metals were used: ρ_Fe_ = 7967 kg/m^3^ and ρ_Cu_ = 8935 kg/m^3^.

### 2.2. Potentials

A description of the interactions in the model was made in a similar manner as the model of the formation of Fe–Cu bimetallic nanoparticles [21] within the embedded atom method (EAM) formalism [32], using the binary EAM of potentials developed in [33] based on the potentials for pure Fe [34] and Cu [35] components.

The embedded atom method is implemented in the software package LAMMPS (Large-scale Atomic/Molecular Massively Parallel Simulator by Sandia National Laboratories, Livermore, CA, USA) [36,37]. The calculations were performed on the Lomonosov-2 supercomputer [38,39] (Lomonosov Moscow State University, Russia) and the supercomputer Zhores (CDISE, Skoltech, Russia) [40].

### 2.3. Cold-Sintering Model

Cold sintering or high-pressure consolidation is a method of near-full-density powder compaction at room or slightly elevated temperature by application of high pressure up to several GPa [11]. Provided that the consolidation temperature is below the melting temperature while taking into account the size-dependent melting point depression of nanoparticles [41], we can obtain a nanostructured composite (nanocomposite).

Earlier on, we experimentally investigated the formation of Fe–Cu nanocomposites by cold sintering of bimetallic nanopowders [42]. Before high pressure consolidation, the samples were treated in a hydrogen flow at 450 °C to remove the surface oxide layer from the nanoparticles.

Pre-simulation. To simulate conditions close to the experimental ones for all model nanopowders, we performed a short pre-simulation: the powder was heated from 300 K to 750 K at constant volume of the simulated area for the duration of 400 ps and then cooled down from 750 K back to 300 K over 500 ps. After that, the powder was kept for 100 ps at the constant temperature T_0_ = 300 K in NVT conditions (N—atom count, V—volume, T—temperature). In all simulations, periodic boundary conditions (PBCs) were specified for all the axes.

Apart from creating conditions that are closer to the experiment, the preliminary simulation stage has other positive aspects. Firstly, in the course of pre-simulation, monometallic F and G particles take on a more energetically favorable shape (non-spherical one) that is defined by the crystal lattice and surface energy of the nanoparticles. Secondly, all nanoparticles significantly shift from the forced initial positions, thus making powder nanoparticles randomly distributed throughout the volume.

**Figure 1:**
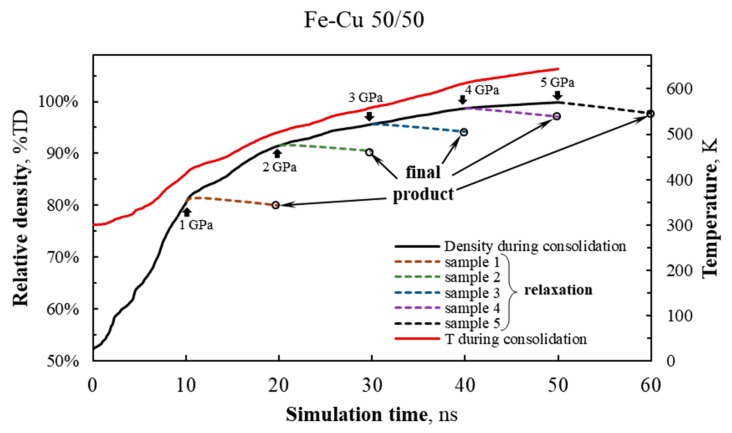
Relative density (solid black line) and temperature (red line) of the 50/50 Fe–Cu nanopowder during cold sintering as a function of simulation time. Pressure in the system is given by *p*(*t*)/GPa = 0.1·*t*/ns, where *t* is time. Density change during sample relaxation to normal conditions (# 1, # 2, …, # 5, obtained at a compaction pressure of 1, 2, …, 5 GPa) is shown by colored dashed lines.

Cold sintering. For each of the seven nanopowder models, comprehensive stress was applied in the simulated periodicity cell up to the maximum of 5 GPa. The pressure in the system was gradually and linearly adjusted *p*[GPa] = *r_p_* × *t*, where *r_p_* = 0.1 GPa/ns—the rate of pressure increase, *t*—the time in ns. Isoenthalpic conditions were maintained in the system, H = const. Figure 1 shows changes in the relative density of the 50/50 nanopowder and its temperature during consolidation at up to 5 GPa (solid lines). The same dependencies for Fe–Cu 28/72 and Fe–Cu 72/28 nanopowders are shown in Figure S2.

Relaxation. Five snapshots of the system state (that include not only the current atomic coordinates but also atomic velocities) were recorded during consolidation simulation at *t* = 10, 20, …, 50 ns (the pressure amounted to *p* = 1, 2, … and 5 GPa, respectively) for further relaxation to normal conditions (T_0_ = 300 K and *p*_0_ = 0.1 MPa) independently of other samples. Relaxation for each sample was performed over 10 ns, and the temperature and pressure in the system were altered linearly. During pressure reduction to *p*_0_ the density of samples was also decreasing (dashed lines in Figure 1 and Figure S2).

As a result, for each nanopowder model several samples compacted at different pressures were produced. For each of the Fe–Cu 28/72, 50/50, and 72/28 nanopowder models, five samples were obtained. Among 21/79, 36/64, 65/35, and 80/20 nanopowder models, relaxation was performed only for samples compacted at 3 GPa and 5 GPa. Thus, for subsequent numerical simulation, we obtained 23 simulated compacts (Table S2).

It is worth mentioning that because of the technical limitations imposed on the atom count, a very fine powder with the average nanoparticles size of <*D*>~140 Å was used in the simulations. The simulated system contains no impurities, in particular oxygen (no oxide films), and the pores are filled with vacuum. This should be taken into account when comparing the simulation estimations with the corresponding experimental results. The advantages and drawbacks of the model are discussed below.

### 2.4. Advantages, Assumptions, and Drawbacks of the Model and Method

Advantages. The advantages of the approach lie in the opportunity to study any geometric and topologic characteristics of the consolidation product on the atomic scale. The advantage of the developed models is the use of initial bimetallic nanoparticles with the Janus structure instead of monometallic particles with simple structure [16,18,43]. These Janus particles were obtained from the simulation of collision and merging of pure Fe and Cu nanoparticles, i.e., the process that actually takes place in the course of bimetallic nanoparticle synthesis using the electrical explosion of wires.

Assumptions. We assume that the selected process model (comprehensive isoenthalpic stressing over 50 ns with subsequent linear cooldown and stress removal over 10 ns) provides a satisfactory simulation of the cold-sintering process. We also assume that the selected EAM potentials provide a rather accurate simulation of the bimetallic system under selected stress conditions.

Drawbacks. A significant downside of the approach lies in the small model size that includes only 16 nanoparticles in a periodicity cell. This hampers us from generalizing the estimations of the topological properties of consolidation products with certain Fe-to-Cu ratios to all cases with the same component ratios. Here, we can only talk about the results obtained for one specific implementation in question. Also, to obtain the target values of the Fe-to-Cu ratio in the 21/79 and 80/20 powder models (i.e., extreme values in terms of component ratios), we had to replace D type bimetallic nanoparticles (see Table 1) with pure monocrystalline F and G type nanoparticles (see Table 2, rows 1 and 7). That is why the results of the analysis of the atomic structure and topological properties of the samples obtained from 21/79 and 80/20 model nanopowders may deviate from the trend that is common among other samples. Furthermore, the size distribution of initial nanoparticles is very narrow: 6% of nanoparticles have a diameter of ~75 Å, 25%~125 Å, and 69%~150 Å. Therefore, all estimated densities and porosities should be generalized to real-life situations with caution.

### 2.5. Methods of Structural and Topological Analysis

#### 2.5.1. Atomic Structure Analysis

Adaptive common neighbor analysis was conducted for all 23 simulated samples (a-CNA) [44,45], and for each atom belonging to crystalline lattice the type of lattice was determined. This method does not determine the structure for atoms located at the surface of solid bodies or at the boundaries with the pore volume. In this research, the analysis is limited to four basic structure phases of Fe–Cu compacts: bcc and fcc lattices, hexagonal close-packed (hcp) lattice, and disordered phase. Other types of lattices in the simulated samples account for under 0.005%. The fourth type (disordered phase) includes both the material in the amorphous state and the atoms located at the boundary with the pore volume. It is worth mentioning that it is not correct to describe atoms located at the pore surface as disordered because locally the surface can be a face of a crystal. However, we will still refer to such atoms as disordered, assuming that the material is not in an ordered/periodic volumetric (bulk) phase.

One drawback of using the method of atomic structure analysis is that for small groups of atoms, as well as for single atoms of one metal (e.g., Cu) occupying the lattice points of the other metal (e.g., Fe), the former metal will be assigned the crystal structure of the host metal. Thus, some atomic groups of Cu in bcc-Fe are identified as bcc-Cu. The same applies to one-atom-thick Cu layers neighboring with bcc-Fe. As this is physically inaccurate, we’ll further refer to such copper phase as quasi-bcc.

#### 2.5.2. Topological Analysis

From the point of view of topological analysis, the studied samples of Fe–Cu compacts are three-dimensional scalar fields, at each point of which, in our case, we can uniquely determine two features from the following set:

- substance type, possible values (“phases”): iron, copper or vacuum (nanopore);
- lattice type determined from the a-CNA results, possible values (“phases”): bcc, fcc, hcp, or disordered (amorphous or surface atoms).

To describe and analyze the structure of such materials, we can use topological invariants, i.e., Minkowski functionals, for each of the “phases.” Initially, in integral geometry, these functionals were restricted to the class of convex bodies. Work [22] provides an extension of the Minkowski functionals definition to non-convex sets such as real spatial structures. Let’s briefly describe the basic definitions of this theory.

Let *X* be a distributed “phase” limited by surface *δX* in the three-dimensional Euclidean space. The four Minkowski functionals of phase X are as follows. *M*_0_(*X*) is the phase volume:

(1) $$M_{0}\left(X\right)=V\left(X\right).$$

*M*_1_(*X*) corresponds to the surface area *δX*:

(2) $$M_{1}\left(X\right)=\underset{\delta X}{∯}dS=S\left(X\right)$$

where *dS* is the surface element. *M*_2_(*X*) is the integral of the mean curvature of surface *δX*:

(3) $$M_{2}\left(X\right)=\underset{\delta X}{∯}\left(\frac{1}{r_{1}}+\frac{1}{r_{2}}\right)dS=C\left(X\right)$$

where *r*_1_ and *r*_2_ are the main surface curvature radii (in a point). *M*_3_(*X*) is the integral of the Gaussian (full) curvature over the phase surface:

(4) $$M_{3}\left(X\right)=\underset{\delta X}{∯}\frac{1}{r_{1}r_{2}}dS=2\pi \chi \left(\delta X\right)=4\pi \chi \left(X\right)=K\left(X\right)$$

where χ(*X*) and χ(*δX*) are the Euler–Poincaré characteristics of body *X*.

The Euler–Poincaré characteristic for a volumetric body is an integral evaluation of its topological complexity and can be defined as a sign-alternating sum of Betti numbers [23,30]:

χ = b_0_ − b_1_ + b_2_,

where b_0_—the number of connected components in the structure, b_1_—the number of end-to-end “holes” in the structure, and b_2_—the number of cavities in the structure. The Euler–Poincaré characteristic is a topological invariant and it does not depend on the geometrical properties of the structure (i.e., the volume and the surface area).

Thus, for each “phase” of the 3-D structure we can determine the following values: volume (or specific volume), (specific) surface area, the integral of mean surface curvature and the Euler–Poincaré characteristic. We can also determine the areas of joint surfaces of the phases in question.

The first three functionals have a simple geometric interpretation and are traditionally used to describe the geometric characteristics of the volumetric structures. But to predict the physical properties of real structures, the first three Minkowski functionals are not enough; in accordance with Hadwiger’s theorem, the system of Minkowski functionals, the last of which is the Euler–Poincaré characteristic, is complete [29]. It means that any φ(*X*) functional that is additive, continuous, and invariant with respect to rotation and shear transformations (including one that characterizes the physical and mechanical properties of the structure) can be represented as a linear combination of Minkowski functional:

(5) $$\phi \left(X\right)=\overset{3}{\underset{i=0}{\sum }}a_{i}M_{i}\left(X\right).$$

Here coefficients *a_i_* preceding the Minkowski functionals can be defined from the experiment or evaluated from the numerical model. Different aspects pertinent to the implementation of Minkowski functional calculations are considered in works [46,47].

This theorem has a fundamental significance for estimating the properties of multi-phase materials defined by the geometrical structure of phases.

## 3. Results and Discussion

### 3.1. Density and Porosity of Cold-Sintered Fe–Cu

**Figure 2:**
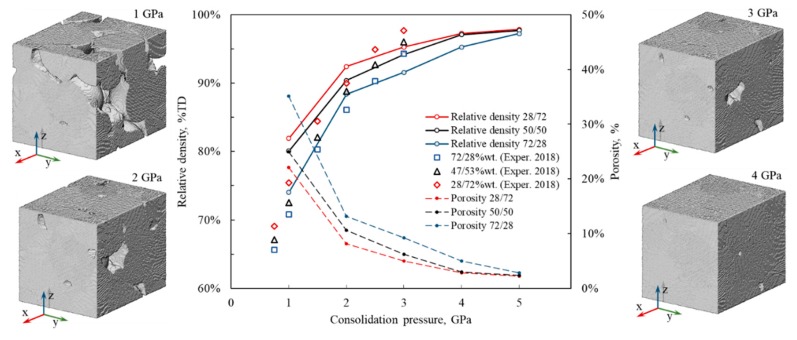
Calculated relative density (solid lines) and porosity (dashed lines) of cold-sintered Fe–Cu 28/72 (red), 50/50 (black), and 72/28 (blue) samples as a function of consolidation pressure. Experimental data points are shown by various symbols [42]. Four images of the model Fe–Cu 50/50 sample obtained at different consolidation pressures are included.

The calculated relative density and porosity of cold-sintered Fe–Cu samples with different Fe contents are shown in Figure 2 as a function of consolidation pressure. As expected, density increases with increasing pressure. For pressures of up to 3 GPa, higher calculated relative densities are achieved for samples with lower Fe contents. At 4 GPa, the relative densities of the Fe–Cu 28/72 and 50/50 samples differ by only 0.6% whereas the density of the 72/28 sample is still noticeably lower. At 5 GPa, comparable densities in the range of 97.2–97.8% are obtained for all the Fe–Cu compositions. A detailed qualitative assessment of the absolute and relative density of simulated samples is provided in Tables S3 and S4.

The calculated densities in Figure 2 are in good agreement with our earlier experimental results of cold sintering of Fe–Cu nanopowders at pressures up to 3 GPa [42]. At 2.5 GPa, the calculated and experimental densities are practically identical. Below this pressure, MD simulations predict RD values slightly higher than the experimental data, whereas above 2.5 GPa the calculated densities are somewhat lower than the experimental ones. At least two flow-related factors could be responsible for this discrepancy. One is the presence of oxide layers on the experimental powders that had not been accounted for in the model. Such surface oxide films are formed even when the ultra-clean iron and copper surfaces are exposed to air at room temperature [48] and can compromise the compressibility of nanopowders. The other factor is the significantly larger (5–10 fold) average diameter of the nanopowders used in the experiment [42] compared to those used in the simulations and the correspondingly different density that can be achieved by these particles at a given pressure.

**Figure 3:**
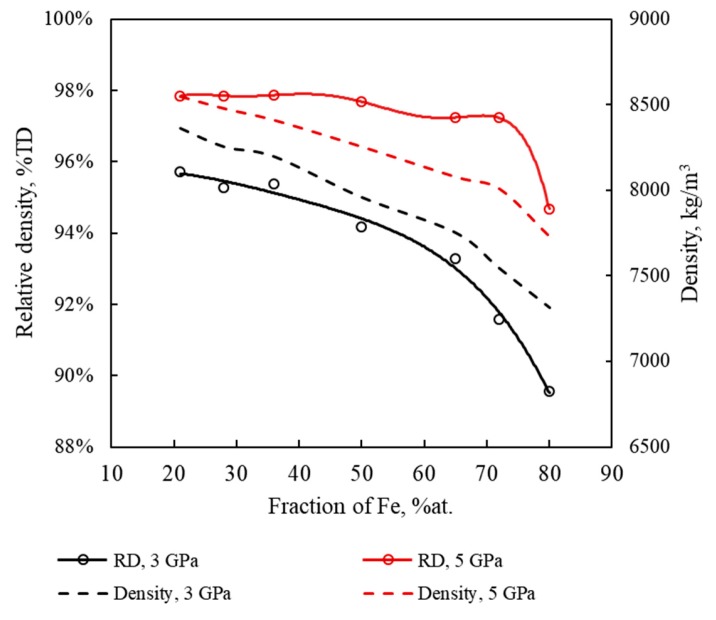
The relative (solid lines) and absolute (dashed lines) density of Fe–Cu samples cold sintered at 3 GPa (black lines) and 5 GPa (red lines) as a function of Fe content.

**Figure 4:**
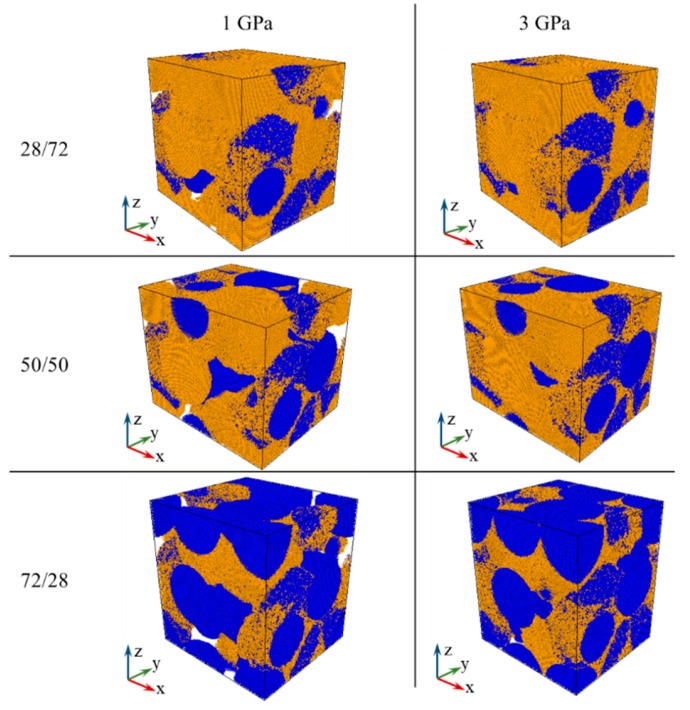
The structure of obtained 28/72, 50/50, and 72/28 Fe (blue)–Cu (orange) samples consolidated at 1 GPa (left column) and 3 GPa (right column). Large unfilled cavities (white) are observed in the samples consolidated at 1 GPa and in the sample with large (72 at.%) content of iron consolidated at 3 GPa (the leftmost sample at the bottom).

Another observation from Figure 2 is that lower relative densities are achieved for Fe–Cu samples with higher Fe (lower Cu) content, again in agreement with the experimental results [42]. The dependence of the simulated relative and absolute density of samples cold sintered at 3 and 5 GPa on iron content is presented in Figure 3. The density decrease with the increase of Fe content is especially pronounced for the sample containing 80 at.% Fe. Figure 4 shows simulated samples with different iron content cold sintered at 1 and 3 GPa. It can be seen that following pressure application, iron segments (blue) of Fe–Cu nanoparticles remain practically undeformed whereas copper fragments (orange) lose their initial shape and penetrate the voids between the particles. It is therefore assumed that the densification of Fe–Cu nanopowder mostly occurs by the plastic flow of the softer copper phase. Large voids are observed in all the samples consolidated at 1 GPa and in the Fe-rich 72/28 sample consolidated at 3 GPa. Apparently, there is not enough copper in the latter sample to fully fill the empty spaces between the relatively rigid grains of iron.

### 3.2. Atomic Structure of Fe–Cu Compacts

**Figure 5:**
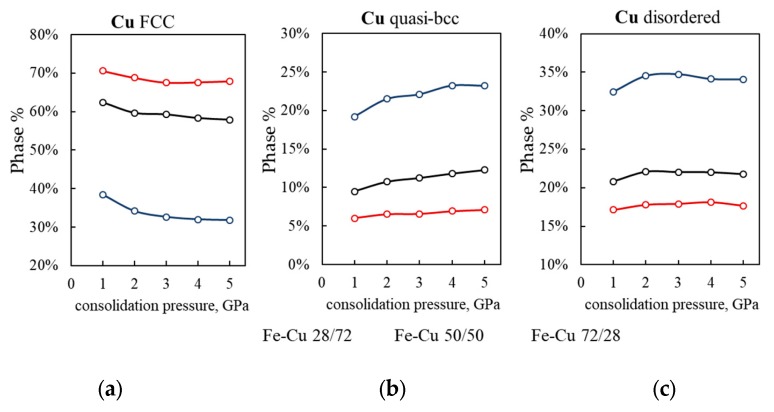
The calculated crystal structure of the Cu component – fcc (**a**), quasi-bcc (**b**) and disordered phase (**c**) as a function of consolidation pressure for three different Fe–Cu compositions: 28/72 (red), 50/50 (black), 72/28 (blue).

Under normal conditions of pressure and temperature, iron has a bcc lattice and copper an fcc lattice [33]. Our MD simulations show that during cold sintering of Fe–Cu nanopowders, significant changes in the crystal structure of copper take place, while iron retains almost entirely its initial bcc lattice. This must be due to the fact that when high pressure is applied, the softer Cu flows around the rigid Fe particles that remain virtually undeformed (Figure 4). The percentage of different phases of Cu present in simulated 28/72, 50/50, and 72/28 Fe–Cu samples cold sintered at different pressures is shown in Figure 5.

**Figure 6:**
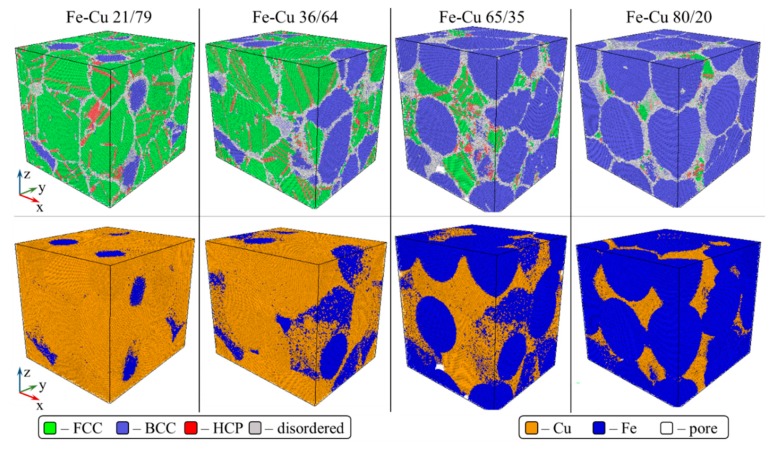
Crystal structure (upper row) and elemental distribution (lower row) of simulated 21/79, 36/64, 65/35, and 80/20 Fe–Cu samples cold sintered at 3 GPa.

**Figure 7:**
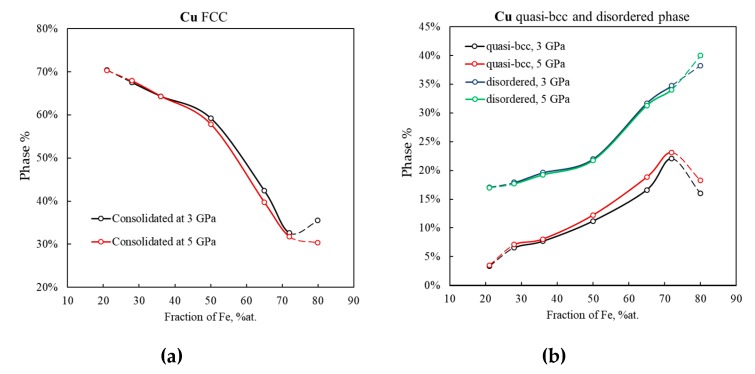
Relative amounts of the fcc phase (**a**) and quasi-bcc and disordered phase (including atoms on pore surface) (**b**) in the copper component of Fe–Cu samples cold sintered at 3 and 5 GPa as a function of Fe content. The rightmost and leftmost parts of the plots are dashed to indicate that the 21/79 and 80/20 Fe–Cu model powders were made up from a different set of nanoparticles (see Section 2.4).

MD simulations reveal three major arrangements of Cu atoms: a regular fcc lattice (Figure 5a), a quasi-bcc lattice (Figure 5b), and a disordered phase (Figure 5c). The relative amount of each phase depends on both consolidation pressure (Figure 5) and Fe-to-Cu ratio (Figure 6 and Figure 7).

#### 3.2.1. Quasi-bcc Copper Phase

As the consolidation pressure increases, the content of the quasi-bcc Cu phase increases and the content of fcc-Cu decreases. This change is greater in samples with the higher content of iron (Figure 5a,b). As the Fe fraction increases from 21% to 80%, the content of the quasi-bcc phase in Cu structure increases from 3.5% to 23% (Figure 7b, black and red lines). The rearrangement of some Cu atoms into a bcc lattice could be a result of the presence of a one-atom thick copper film on the surface of the initial quasi-Janus Fe–Cu nanoparticles. During high pressure consolidation, this thin Cu film is confined between bcc-iron surfaces which favors the bcc atomic arrangement in such Fe–Cu–Fe nanolayers. This special feature of cold-sintered bimetallic Fe–Cu nanoparticles is hardly observed during consolidation of elemental Fe and Cu powder blends. The larger the Fe/Cu ratio in the bimetallic nanoparticles, the larger the surface area of iron and the number of Cu atoms located on the Fe surface. This explains the larger relative amount of quasi-bcc Cu in cold-sintered material with high iron content (Figure 5b, compare blue and red curves, and Figure 7b).

It is worth noting that several experimental reports on the metastable bcc-Cu have been published. Epitaxial growth of thin Cu films with bcc structure on the Fe {001} surface was reported in [49]. The existence of 2–3-atomic-layer thick bcc-Cu films and the coexistence of the bcc and fcc phases in 10–12-atomic-layer thick Cu films were observed on the Ag {001} surface [50]. Cu precipitates with bcc structure were experimentally observed in iron and steels [51,52]. The possibility of fcc-to-body centered phase transformation under shock loading of Cu single crystal was also demonstrated using computer simulations [53,54]. In one theoretical study [55], ab initio calculations based on the cluster expansion method were performed to demonstrate that the bcc phase of Fe_1−x_Cu_x_ solid solutions with >50 at.% Cu has a negative shear modulus and is therefore mechanically unstable. This is in agreement with our observations of decreasing quasi-bcc Cu content with decreasing Fe-to-Cu ratio (Figure 7b, black and red lines).

#### 3.2.2. The Disordered and Hcp Phases

The analysis of crystal structure of the simulated Fe–Cu samples shows that along with the fcc and bcc phases, regions with a disordered atomic arrangement are present at Cu–Cu and Fe–Fe grain boundaries, as well as at Fe–Cu interfaces (Figure 6, gray).

For all the compositions, the content of the disordered phase increases with pressure up to 2 GPa and remains constant at pressures above (Figure 5c). The higher the content of Fe in the nanopowder, the larger the percentage of disordered copper. This is because for the higher content of the Fe phase its surface area is also larger and the space not occupied by iron has a more tortuous and complex shape. Plastic flow of copper into this space generates high shear stresses causing copper to undergo considerable phase transformations. As a result, the fcc phase ratio in the copper component of 80/20 Fe–Cu samples can be as low as 30% (vs. 70% for the 21/79 sample) (Figure 7a), and the disordered phase ratio as high as 40% (vs. 17% for the 21/79 sample) (Figure 7b).

In addition to the phases discussed above, a small amount (7.5–11.5%) of hcp-Cu is present in the simulated samples, the highest value corresponding to the maximum Fe content (80%) and the highest consolidation pressure (5 GPa). As seen from the a-CNA results in Figure 6 (upper row), the hcp phase appears as flat, 1–2 (seldom 3–4) atomic-layers-thick bands (red) that interrupt the regular stacking sequence of layers in fcc-Cu (green) (Figure S3).

The formation of hcp stacking faults in copper, as well as amorphization at Cu–Nb (fcc–bcc) interfaces were previously observed in MD simulations of severe plastic deformation of an immiscible Cu–Nb system [56]. An amorphous layer between Cu and Nb was also observed experimentally in Cu–Nb wires fabricated by cold drawing with a large strain [57].

### 3.3. Topological Analysis

#### 3.3.1. Minkowski Functionals

**Figure 8:**
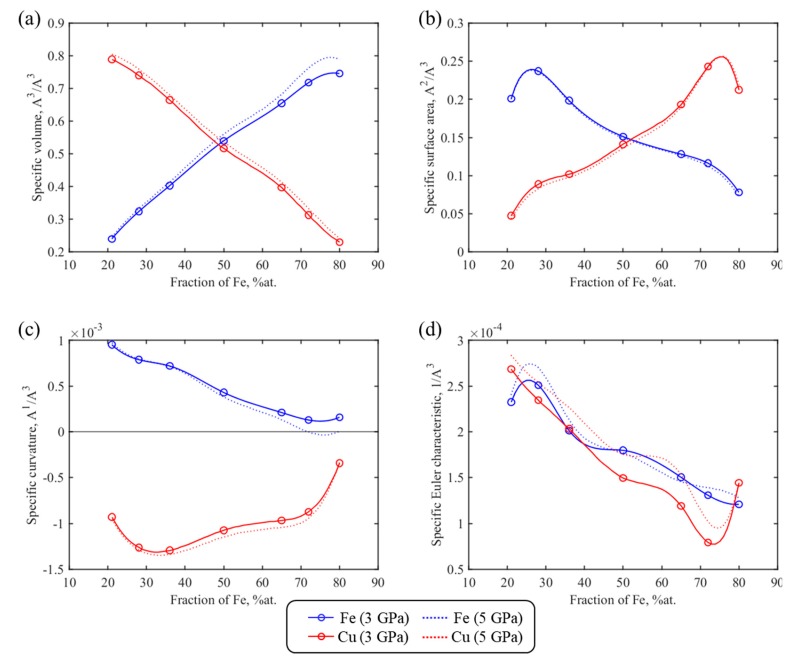
Four Minkowski functionals for Fe (blue) and Cu (red) phases as a function of Fe fraction in cold-sintered samples *M*_0_ (**a**), *M*_1_ (**b**), *M*_2_ (**c**), and M_3_ (**d**). The results are presented for two consolidation pressures: 3 GPa (solid lines) and 5 GPa (dotted lines).

In Figure 8, calculated Minkowski functionals of the Fe and Cu components of Fe–Cu samples cold sintered at 3 and 5 GPa are shown as a function of Fe content. The M_0_ functionals (volume) change linearly as the Fe content of the nanocomposite increases: M_0_(Fe) goes up and M_0_(Cu) goes down (Figure 8a).

While considering the dependence of topological characteristics on input parameters, a note should be made that our MD simulations of cold-sintered Fe–Cu samples revealed the existence of both large Fe and Cu grains with small curvature and specific surface area, and of single atoms and clusters of one metal in the other, having high topological characteristics. There are more single atoms of Fe in the Cu matrix than single atoms of Cu in the Fe matrix. For example, for the 50/50 Fe–Cu sample consolidated at 3 GPa, the difference is three-fold. The higher the metal (Fe or Cu) content in the simulated sample, the smaller the proportion of single-atom clusters of this metal and the correspondingly lower the functional *M*_1_ corresponding to the specific surface (Figure 8b). Furthermore, as discussed above, when high pressure is applied copper flows into the free space between iron particles. As the content of Fe in the sample increases, this space becomes a more tortuous and complex shape, which also leads to the enhancement of the specific surface area of the copper phase (Figure 8b, red line).

As the content of iron goes up, the absolute value of functionals *M*_2_(Fe) and *M*_2_(Cu) decreases. For all the Fe–Cu compositions, the functional *M*_2_(Fe) has a positive value suggesting the convex shape of Fe grains (Figure 8c). The specific surface curvature of Cu phase is negative (Figure 8c, red line), which corresponds to the large number of concave Cu surfaces in contact with the convex iron grains.

The dependences of Minkowski functionals for the Fe and Cu phases on consolidation pressure are presented in Figures S4 and S5 for three different Fe–Cu compositions: 28/72, 50/50, and 72/28. The results and description of Minkowski functionals for the fcc, bcc, hcp lattices, and the disordered phase as a function of nanopowder composition and consolidation pressure are provided in Supplementary Information (Figures S6–S13).

The estimated Minkowski functionals form a complete basis for formalizing the structure– properties relationship of bimetallic Fe–Cu material using Hadwiger’s theorem (see Section 3.4).

#### 3.3.2. Pairwise Interfaces

Structural characteristics of multi-phase materials such as, for example, interphase boundary area, strongly impact their physical and mechanical properties, e.g., thermal and electrical conductivities. Computer simulations allow us to quantitatively estimate the area of various internal interfaces, homogeneous (grain boundaries, twin boundaries), as well as heterogeneous (bi-material interfaces or precipitate–matrix boundaries). In the case of a two-component Fe–Cu system, three types of heterogeneous boundaries are present: (1) pore–Fe, (2) pore–Cu interface, and (3) bi-material Fe–Cu interface. In this work, we studied the change of pairwise interface areas with variation of consolidation pressure for three different Fe–Cu compositions: 28/72, 50/50, and 72/28 (Figure S14). The simulation results have shown that as the pressure increases from 1 to 5 GPa, the specific areas of the pore–Fe (Figure S14a) and pore–Cu (Figure S14b) interfaces decrease manyfold, while the Fe–Cu interface area increases (Figure S14c), presumably due to the decrease in the pore volume. It must be noted that in the 28/72 and 50/50 samples, the area of the pore–Cu interfaces is significantly greater than the area of the pore–Fe interfaces, suggesting that the surface of voids is formed predominantly by copper atoms. This could be the consequence of the thin Cu crust initially present on the surface of many bimetallic nanoparticles that could be maintained in the course of cold sintering. Moreover, as the surface energy of copper is lower than that of iron, the formation of a new (secondary) Cu crust could occur at the elevated temperatures recorded during consolidation.

#### 3.3.3. Mean Phase Thickness

Here we use two Minkowski functionals, *M*_0_(*X*) to *M*_1_(*X*), to introduce an additional useful topological characteristic, namely “mean phase thickness”. Let’s consider a test object with the shape of parallelepiped whose thickness *w*(P) is negligible compared to its length *l* and height *h* (*w* << *h*, *l*). The volume of such parallelepiped is *V* = *h* × *l* × *w* and its surface area can be written as *S* = *2h* × *l*. Given that V = *M*_0_(*X*) and S = *M*_1_(*X*), the expression for the thickness *w* will be:

(6) $$w\left(X\right)=2\frac{V}{S}=2\frac{M_{0}\left(X\right)}{M_{1}\left(X\right)}.$$

In other words, the ratio of the Minkowski functionals *M*_0_(*X*) and *M*_1_(*X*) is an estimate of the “mean thickness” of phase *X*, *w*(*X*). For test objects with simple geometries, e.g., a sphere with diameter D and a cube with edge A, the introduced value *w* will be D/3 and A/3, respectively.

**Figure 9:**
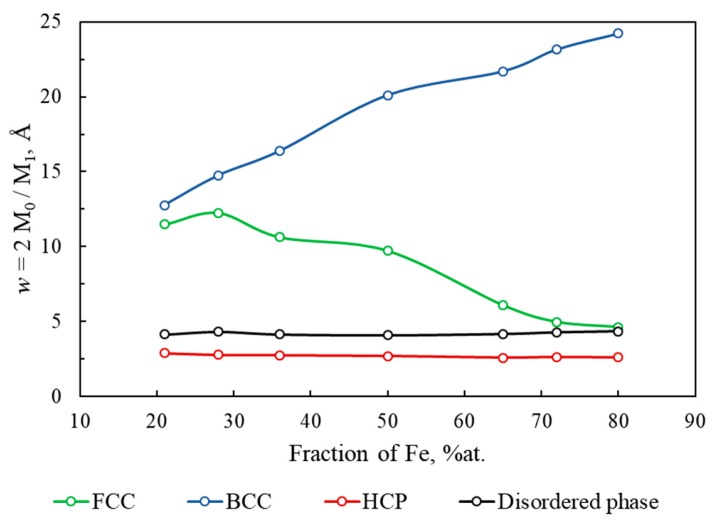
The dependence of mean thickness *w*(*X*) of the phase for fcc (green), bcc (blue), hcp (red) and disordered phase (black) on the iron fraction in the Fe–Cu nanocomposite.

In Figure 9, *w*(*X*) of different phases present in simulated Fe–Cu nanocomposite is shown as a function of iron content. It can be seen that the hcp phase (red line) has the lowest value of *w*(HCP) ≈ 2.7 Å that is independent of Fe content. This is quite reasonable given the results in Figure 6 showing that the hcp phase is distributed as stacking faults two atomic layers thick. The thickness of the disordered phase (black line) is only slightly larger (*w* ≈ 4.2 Å) and is also independent of the sample composition. This is again in agreement with phase distribution in Figure 6 where the disordered phase appears as a several-atoms-thick layer between Fe and Cu regions or grains (crystallites) or between differently oriented same metal grains.

The thickness of the bcc phase, *w*(BCC) (blue line), increases with increasing Fe content, suggesting that the greater part of iron exists as large grains with small surface area. *w*(FCC) (green line) decreases with increasing Fe content (decreasing Cu content) and becomes comparable with the mean thickness of the disordered phase in the most iron-rich 80/20 sample.

### 3.4. A New Tool for Computer-Aided Design of Fe–Cu Nanocomposites

Based on Hadwiger’s theorem and the obtained topological invariants, we propose here a new tool for computer-aided design of Fe–Cu nanocomposites.

Consider a physical property P of a material which satisfies the conditions of Hadwiger’s theorem, i.e., is additive, continuous, and motion invariant. In addition to density, the property P could be, within a certain accuracy, thermal and electrical conductivity, compressibility, elastic modulus, Poisson’s ratio, heat capacity, thermal expansion coefficient, etc. For the property P of a bimetallic Fe–Cu material, the relation (5) can be rewritten as:

(7) $$P\left(p,r\right)≅\overset{3}{\underset{i=0}{\sum }}a_{i}^{Fe}M_{i}^{Fe}\left(p,r\right)+\overset{3}{\underset{i=0}{\sum }}a_{i}^{Cu}M_{i}^{Cu}\left(p,r\right)$$

where $a_{i}^{Fe}$ and $a_{i}^{Cu}$ are eight unknown coefficients (*i* = 0, 1, 2, 3), and *p* and *r* are the consolidation pressure and Fe-to-Cu ratio, respectively. At this stage we have estimated eight Minkowski functionals $M_{i}^{Fe}\left(p,r\right)$ and $M_{i}^{Cu}\left(p,r\right)$ at 23 different points of the *p–r* domain that constitute the first 184 units in the created functionals library (see Section 3.3.1 and Supplementary Information).

**Scheme 1:**
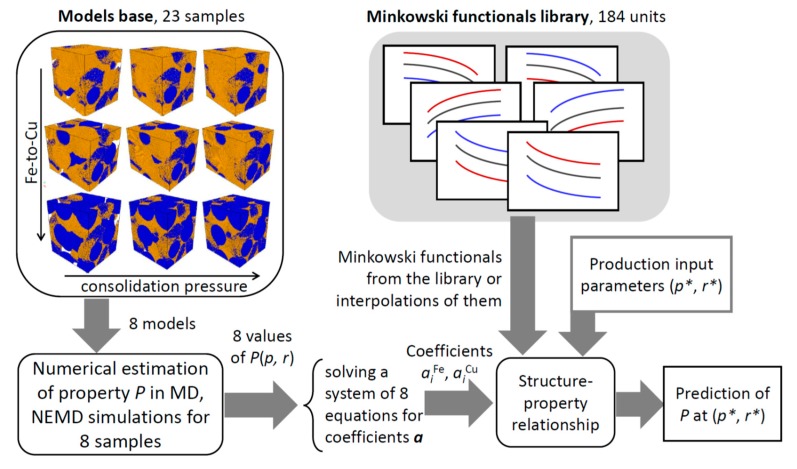
Property prediction approach based on the obtained topological invariants and Hadwiger’s theorem.

To formalize the relationship between structure and property P, it is first necessary to estimate this property in eight different points of the p–r space. This can be done using any eight (or more) of the 23 models listed in the “Models base” and any relevant numerical method (classical or non-equilibrium MD) (see Scheme 1). Then the coefficients $\;a_{i}^{Fe}$ and $a_{i}^{Cu}$ can be found by solving the system of eight equations following from the relation (7) (or by minimization of the equation error, if more than eight points have been chosen):

(8) $$\begin{array}{c} \underset{i}{\sum }a_{i}^{Fe}M_{i}^{Fe}\left(p_{1},r_{1}\right)+\underset{i}{\sum }a_{i}^{Cu}M_{i}^{Cu}\left(p_{1},r_{1}\right)=P\left(p_{1},r_{1}\right),\\ \ldots \\ \underset{i}{\sum }a_{i}^{Fe}M_{i}^{Fe}\left(p_{8},r_{8}\right)+\underset{i}{\sum }a_{i}^{Cu}M_{i}^{Cu}\left(p_{8},r_{8}\right)=P\left(p_{8},r_{8}\right). \end{array}$$

Once the coefficients are known, an explicit mathematical expression (7) that approximates the property P of Fe–Cu nanocomposites as a function of consolidation pressure *p* and Fe-to-Cu ratio *r* can be written. Compared with the traditional method of approximation of a function of two variables, the proposed approach has a significantly higher accuracy.

Furthermore, the absolute value of coefficients $\;a_{i}^{Fe}$ and $a_{i}^{Cu}$ is indicative of the relative importance of each phase and of the topological characteristic associated with the corresponding Minkowski functional (phase volume, interface curvature, etc.) for a given property P of a bimetallic nanocomposite (see an example for mass density in Supplementary Section S6). The sign of $\;a_{i}^{Fe}$ and $a_{i}^{Cu}$ indicates the direction of property change (increase, decrease). This allows the identification of key structural parameters that should be tuned in order to design a nanocomposite with desired properties.

Alternatively, a property P of any nanocomposite produced by cold sintering of bimetallic Fe–Cu nanoparticles with Fe-to-Cu ratio 0.25 ≤ *r* ≤ 4.0 at consolidation pressure 1 ≤ *p* ≤ 5 GPa can now be determined. If the initial cold-sintering parameters are among the 15 unused *p–r* pairs for which the Minkowski functionals have been determined (out of the total 23 in the library), the property P is estimated by substituting the corresponding $\;M_{i}^{Fe}\left(p,r\right)$ and $M_{i}^{Cu}\left(p,r\right)$ into equation (7). In all the other cases interpolated values of Minkowski functionals can be used. A data flow diagram of the proposed approach is presented in Scheme 1.

## 4. Conclusions

Computer-aided analysis of cold sintering (high pressure consolidation) of bimetallic nanoparticles composed of two immiscible metals—Fe and Cu—was performed using atomistic molecular dynamics simulations and the embedded atom method. The effect of cold-sintering parameters on structure, density, and topology of obtained Fe–Cu nanocomposites was comprehensively studied within the limitations of the numerical model used. The density and porosity of simulated samples were estimated as a function of Fe content and consolidation pressure. The obtained dependences are in a good agreement with the experimental data.

Our MD simulation revealed that the atomic structure of iron remains practically unchanged during high pressure application whereas the copper component undergoes a variety of structural changes, to the extent depending on the Fe–Cu ratio in the initial bimetallic nanopowder.

The Minkowski functionals of the studied system were calculated and their dependence on consolidation pressure and elemental composition of the nanopowder was found. To the best of our knowledge this is the first reported attempt to apply topological analysis using the Minkowski functionals to structure characterization of bimetallic nanocomposites.

Based on the obtained topological invariants and Hadwiger’s theorem, we were able to formalize the relationship between the structure and properties of bimetallic Fe–Cu nanocomposites. The formalized functional relationship can be used as a structure–properties prediction tool for bimetallic nanocomposites, thus contributing to the development of materials by computer-aided design.

The results obtained in this study were used to generate an open-source base of atomistic models of Fe–Cu samples obtained under different cold-sintering conditions and to create a library of the corresponding Minkowski functionals. The data is available for download at the Tsukanov Lab webpage (https://tsukanov-lab.moy.su or at https://csmlab.ru) of the Complex Systems Modeling Laboratory, Lomonosov Moscow State University.

## Figures and Tables

**Table 1:** A set of seven model nanoparticles used for building the models of initial Fe–Cu powders. Model bimetallic A–E nanoparticles were obtained in work [21].

Code	Particle Type	Atom Count (Particle Diameter, Å)	Fe, at.%	Production Method
A	Capped Janus	87,532 (~125)	89.0	Merging a 120 Å Fe nanoparticle with a 60 Å Cu nanoparticle at T = 1500 K
B	Partially engulfed Janus with copper crust	86,150 (~125)	11.3	Merging a 60 Å Fe nanoparticle with a 120 Å Cu nanoparticle at T = 1500 K
C	Acorn-shaped Janus	154,364 (~150)	50.5	Merging a 120 Å Fe nanoparticle with a 120 Å Cu nanoparticle at T = 1500 K
D	Two-hemisphere spherical Janus with copper crust	154,364 (~150)	50.5	Merging a 120 Å Fe nanoparticle with a 120 Å Cu nanoparticle at T = 2000 K
E	Spherical Janus with a clear boundary and copper crust	19,318 (~75)	50.3	Collision of monometallic 60 Å nanoparticles at the relative velocity of 20 m/s and initial T_0_~1680 K
F	Spherical Fe (100%) nanoparticle	151,669 (150)	100	Cutting out a 150 Å diameter sphere from a bulk bcc monocrystal with a = 2.855 Å
G	Spherical Cu (100%) nanoparticle	149,621 (150)	0.0	Cutting out a 150 Å diameter sphere from a bulk fcc monocrystal with a = 3.615 Å

**Table 2:** Model Fe–Cu nanopowders used in the simulations. Different Fe/Cu ratios were obtained by mixing A to G nanoparticles in different proportions.

Model Code	Atom Count	Nanopowder Composition	Fe, at.%.	TD, kg/m^3^
21/79	2 026 742	A_2_B_2_C_2_DEG_8_	20.7%	8737
28/72	2 036 228	A_2_B_2_C_2_D_3_EG_6_	28.2%	8665
36/64	2 038 276	A_2_B_2_C_2_D_3_EFG_5_	35.6%	8593
50/50	2 042 372	A_2_B_2_C_2_D_3_EF_3_G_3_	50.4%	8450
65/35	2 046 468	A_2_B_2_C_2_D_3_EF_5_G	65.1%	8308
72/28	2 048 516	A_2_B_2_C_2_D_3_EF_6_	72.5%	8236
80/20	2 043 126	A_2_B_2_C_2_DEF_8_	79.9%	8164

## Supplementary Materials

The following are available online at https://www.mdpi.com/1996-1944/13/3/541/s1, Figure S1: view of the Fe–Cu 50/50 nanopowder model in two projections; Figure S2: relative density and temperature of nanopowders in the process of cold sintering; Figure S3: hcp–fcc stacking fault in the Cu phase; Figure S4: Minkowski functionals for Fe as a function of consolidation pressure; Figure S5: Minkowski functionals for Cu as a function of consolidation pressure; Figure S6: Minkowski functionals for the fcc phase as a function of iron content; Figure S7: Minkowski functionals for the fcc phase as a function of consolidation pressure; Figure S8: Minkowski functionals for the bcc phase as a function of iron content; Figure S9: Minkowski functionals for the bcc phase as a function of consolidation pressure; Figure S10: Minkowski functionals for the hpc phase as a function of iron content; Figure S11: Minkowski functionals for the hcp phase as a function of consolidation pressure; Figure S12: Minkowski functionals for the disordered phase as a function of Fe content; Figure S13: Minkowski functionals for the disordered phase as a function of consolidation pressure; Figure S14: areas of pairwise pore–iron, pore–copper and iron–copper interfaces as a function of consolidation pressures; Figure S15: case of Fe–Cu composite with a simple geometry and Minkowski functionals for Fe and Cu phases. Table S1: elemental composition of considered Fe–Cu nanopowder models; Table S2: a grid of simulated models of Fe–Cu compacts; Table S3: resultant density estimation of Fe–Cu nanocomposites as a function of consolidation pressure; Table S4: resultant density estimation of Fe–Cu nanocomposites as a function of iron content in the initial nanopowder; Table S5: coefficients a of the expansion of the absolute density ρ of the Fe–Cu nanocomposite in series of Minkowski functionals.
