# Supplementary material for: Effect of Cold-Sintering Parameters on Structure, Density, and Topology of Fe–Cu Nanocomposites
Source: Materials (Basel). 2020 Jan 23;13(3):541. doi: 10.3390/ma13030541 (PMC7040682; doi:10.3390/ma13030541)
Supplement: Supplementary file 1 [file materials-13-00541-s001.pdf]

materials

MDPI

Supplementary Information

Effect of Cold-Sintering Parameters on Structure, Density, and Topology of Fe–Cu Nanocomposites

Alexey Tsukanov [1,*], Dmitriy Ivonin [2], Irena Gotman [3], Elazar Y. Gutmanas [4], Eugene Grachev [2], Aleksandr Pervikov [5] and Marat Lerner [5]

1 Center for Computational and Data-Intensive Science and Engineering (CDISE), Skolkovo Institute of Science and Technology (Skoltech), 30, bld. 1, Bolshoy Boulevard, Moscow 121205, Russia
2 Faculty of Physics, Lomonosov Moscow State University, GSP-1, 1-2 Leninskie Gory, Moscow 119991, Russia; ivonin.dmitriy@physics.msu.ru (D.I.); gracheve a@gmail.com (E.G.)
3 Department of Mechanical Engineering, ORT Braude College, Karmiel 2161002, Israel; irenag@braude.ac.il
4 Department of Materials Science and Engineering, Technion-Israel Institute of Technology, Haifa 32000, Israel; gutmanas@technion.ac.il
5 Institute of Strength Physics and Materials Science of SB RAS, 2/4, pr. Akademicheskii, Tomsk 634055, Russia; pervikov@list.ru (A.P.); lerner@ispms.tsc.ru (M.L.)
* Correspondence: a.a.tsukanov@yandex.ru

Received: 15 November 2019; Accepted: 19 January 2020; Published: date

1. Powder Models

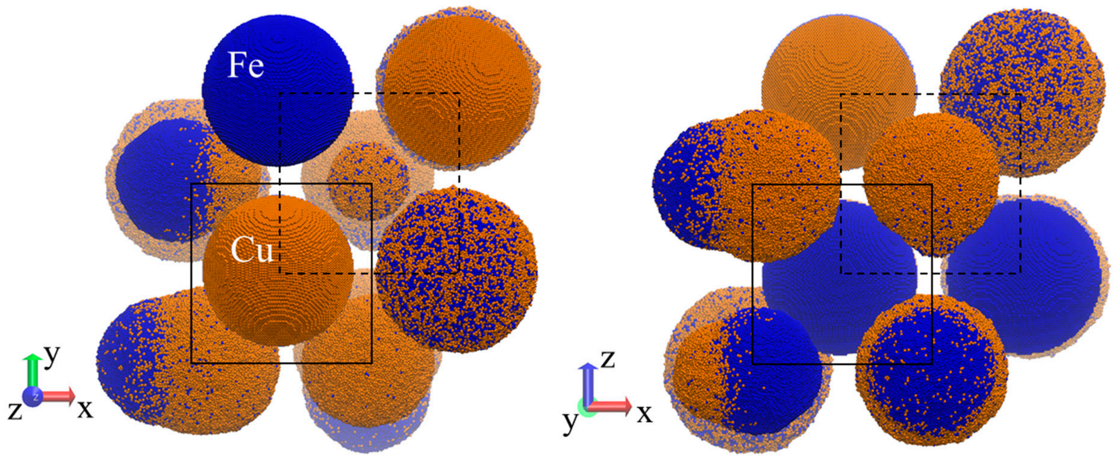

Figure S1. View of the Fe-Cu 50/50 nanopowder model in two projections. Outlines show an elementary cell of an abstract bcc lattice with a = 180 Å in whose nodes nanoparticles are placed. The initial size of the periodic simulated area is $X_0 = Y_0 = Z_0 = 2a$ = 360 Å. Colors: Fe – blue, Cu – orange.

Table S1. Elemental composition of considered Fe-Cu nanopowder models.

Model code	Fe atom count	Cu atom count	Fe, %at.	Cu, %at.	Fe, %wt.	Cu, %wt.
21/79	418 838	1 607 904	20.7%	79.3%	18.6%	81.4%
28/72	574 708	1 461 520	28.2%	71.8%	25.7%	74.3%
36/64	726 377	1 311 899	35.6%	64.4%	32.7%	67.3%
50/50	1 029 715	1 012 657	50.4%	49.6%	47.2%	52.8%
65/35	1 333 053	713 415	65.1%	34.9%	62.2%	37.8%
72/28	1 484 722	563 794	72.5%	27.5%	69.8%	30.2%
80/20	1 632 190	410 936	79.9%	20.1%	77.7%	22.3%

2. Cold Sintering Simulations

(a)

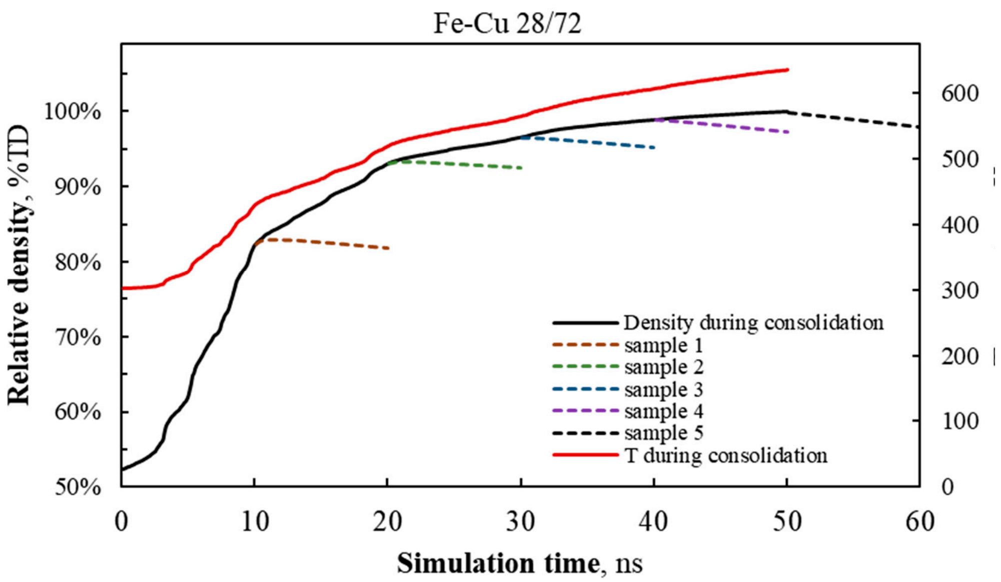

(b)

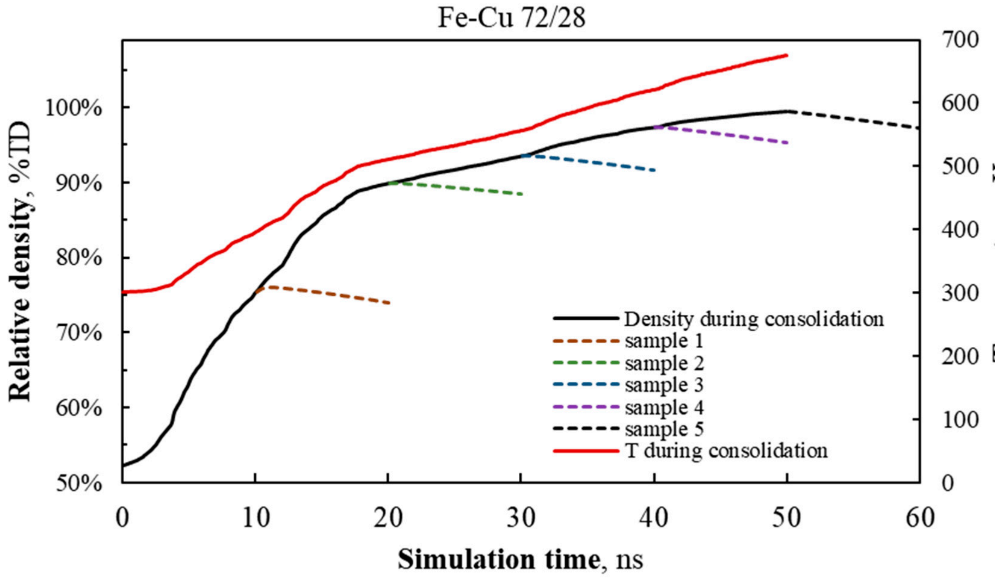

Figure S2. Relative density (solid black line) and temperature (red line) of Fe-Cu 28/72 (**a**) and Fe-Cu 72/28 nanopowders (**b**) in the process of cold sintering at the loading rate of 0.1 GPa/ns. The drift of the density during the relaxation of samples obtained at the consolidation pressures of 1, …, 5 GPa (samples 1, …, 5) to the normal conditions are shown in colored dashed lines.

Table S2. A grid of simulated models of Fe-Cu compacts.

Powder model	1 GPa	2 GPa	3 GPa	4 GPa	5 GPa
21/79	.	.	X	.	X
28/72	X	X	X	X	X
36/64	.	.	X	.	X
50/50	X	X	X	X	X
65/35	.	.	X	.	X
72/28	X	X	X	X	X
80/20	.	.	X	.	X

3. Absolute and Relative Density of Fe-Cu Samples

Table S3. Resultant density estimation of Fe-Cu nanocomposites as a function of consolidation pressure (for three compositions).

P, GPa	Fe-Cu 28/72		Fe-Cu 50/50		Fe-Cu 72/28	
	ϱ, kg/m³	RD, %	ϱ, kg/m³	RD, %	ϱ, kg/m³	RD, %
1.0	7097	81.9%	6765	80.1%	6096	74.0%
2.0	8009	92.4%	7639	90.4%	7279	88.4%
3.0	8254	95.3%	7958	94.2%	7542	91.6%
4.0	8428	97.3%	8204	97.1%	7846	95.3%
5.0	8478	97.8%	8255	97.7%	8008	97.2%

Table S4. Resultant density estimation of Fe-Cu nanocomposites as a function of iron content in the initial nanopowder (for two consolidation pressures).

Fe fraction, %at.	3 GPa		5 GPa	
	ϱ, kg/m³	RD, %	ϱ, kg/m³	RD, %
20.7	8363	95.7%	8549	97.8%
28.2	8254	95.3%	8478	97.9%
35.6	8197	95.4%	8410	97.9%
50.4	7958	94.2%	8255	97.7%
65.1	7751	93.3%	8078	97.2%
72.5	7542	91.6%	8008	97.2%
79.9	7312	89.6%	7729	94.7%

4. Appendix to the Atomic Structure of Fe-Cu Nanocomposite Section

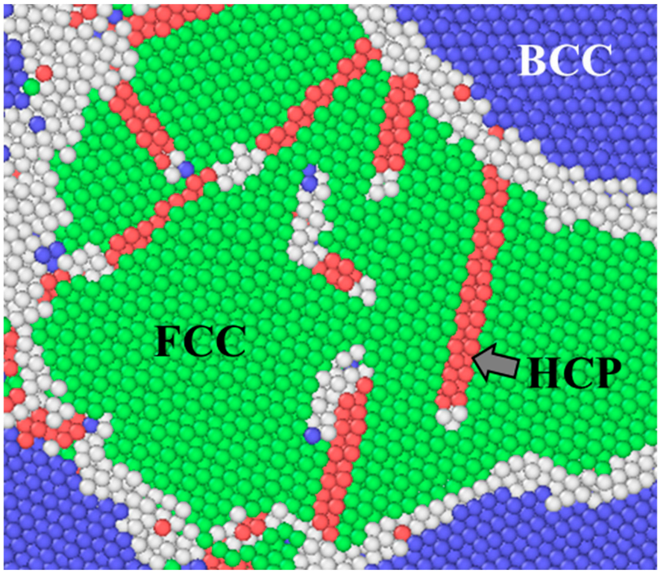

Figure S3. HCP-FCC stacking fault in the Cu phase. Colors: green – FCC, blue – BCC, red – HCP, white – disordered phase.

5. Minkowski Functionals Library

The source data used to plot all the dependencies of the Minkowski functionals on the parameters of cold sintering can be found at Tsukanov-Lab webpage https://tsukanov-lab.moy.su or via link on the cloud drive https://yadi.sk/d/1TdJHckC0XlKNg, and at site https://csmlab.ru of Complex Systems Modeling Laboratory, Lomonosov MSU.

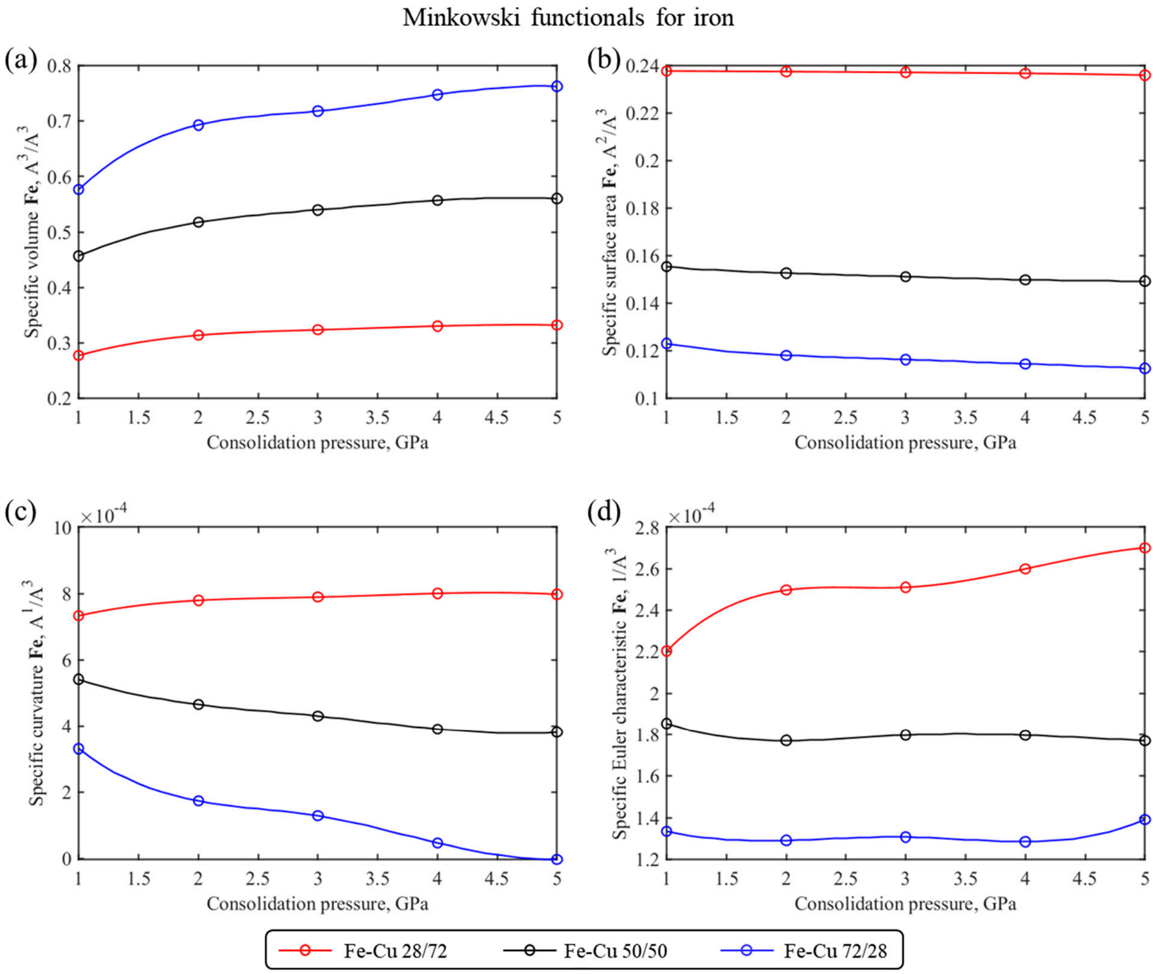

Figure S4. Minkowski functionals for Fe as a function of consolidation pressure: (**a**) M_0(Fe), (**b**) M_1(Fe), (**c**) M_2(Fe), (**d**) M_3(Fe). The results are presented for three different compositions of consolidated Fe-Cu 28/72 (red), 50/50 (black) and 72/28 (blue) samples.

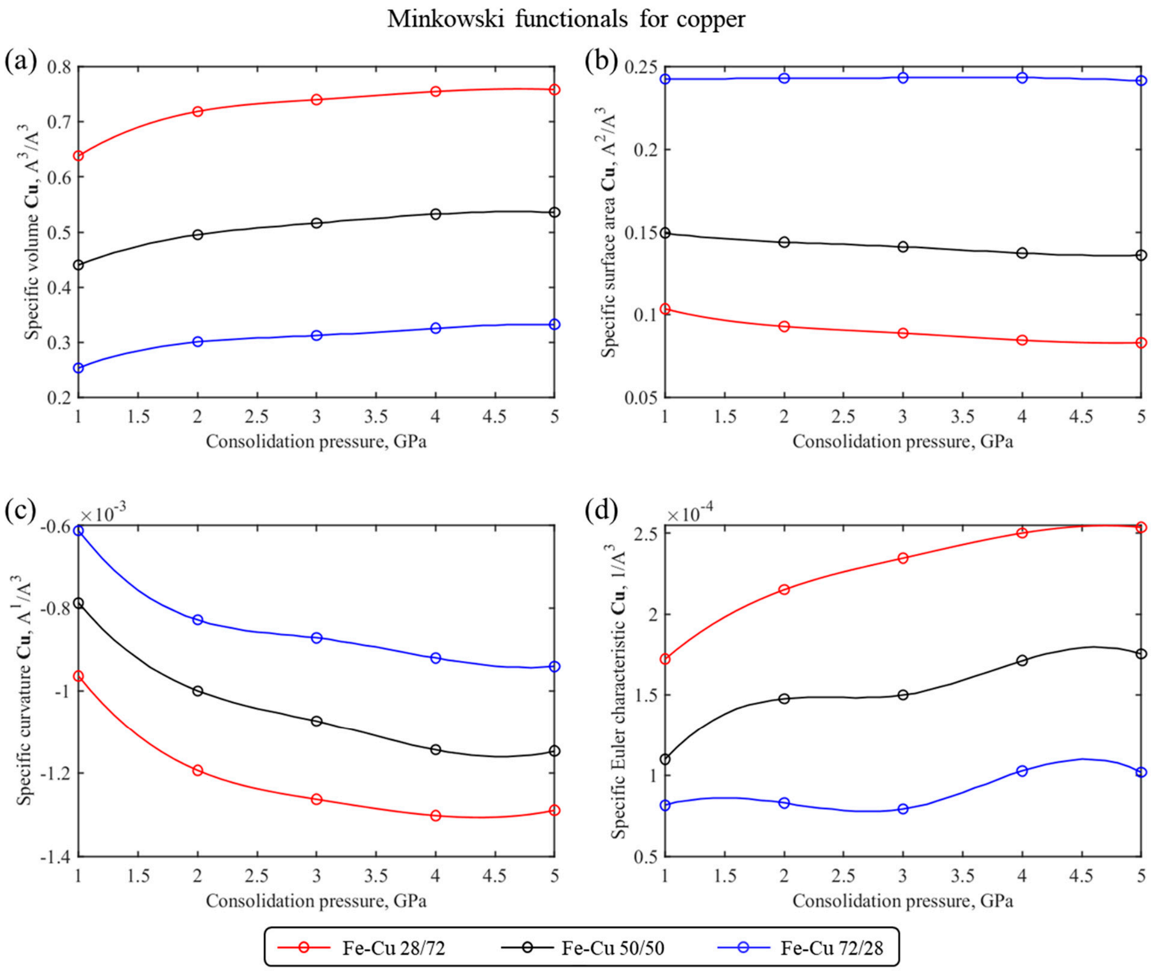

Figure S5. Minkowski functionals for Cu as a function of consolidation pressure: (**a**) M_0(Cu), (**b**) M_1(Cu), (**c**) M_2(Cu), (**d**) M_3(Cu). The results are presented for three different compositions of consolidated Fe-Cu 28/72 (red), 50/50 (black) and 72/28 (blue) samples.

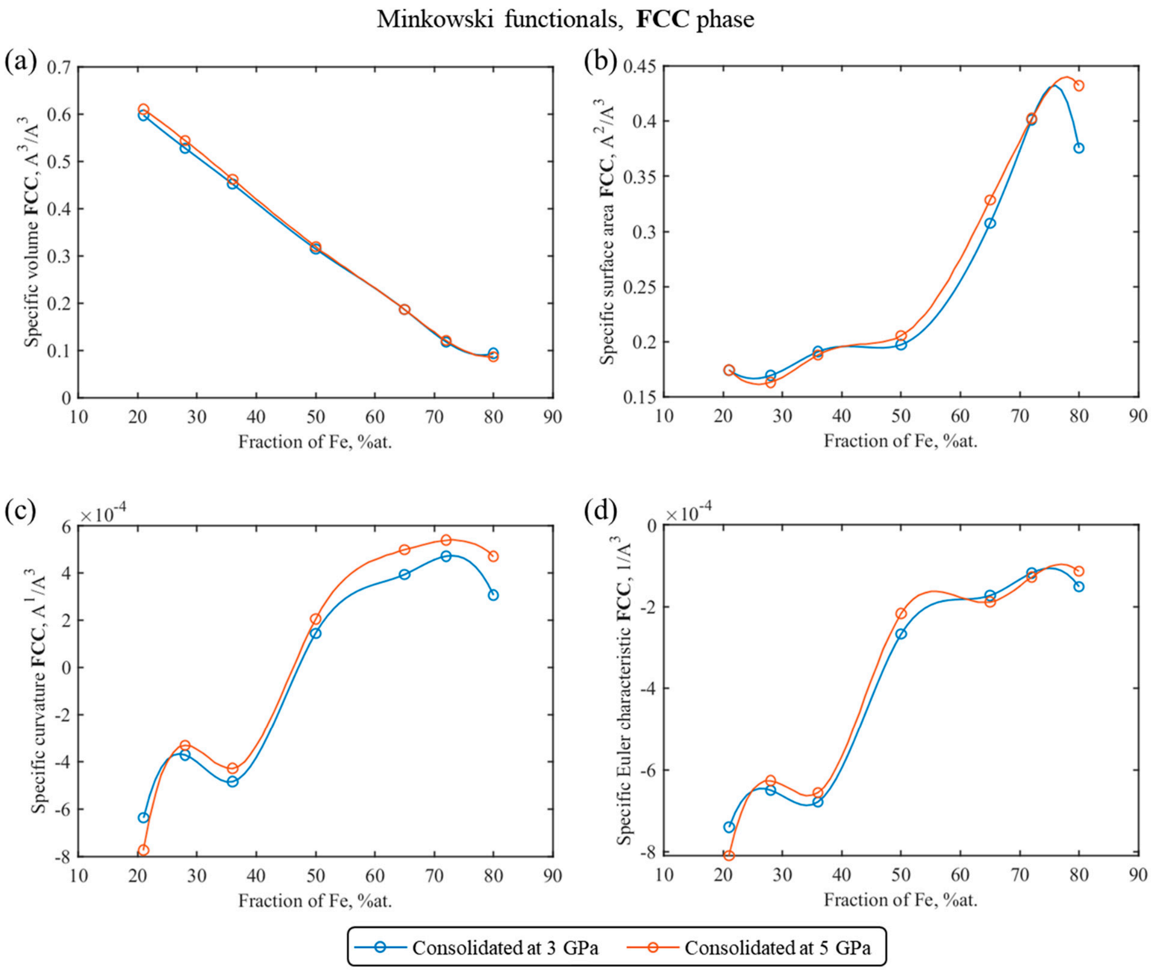

Figure S6. Minkowski functionals for the fcc phase as a function of iron content in consolidated samples: (**a**) M_0(FCC), (**b**) M_1(FCC), (**c**) M_2(FCC), (**d**) M_3(FCC). The results are presented for two consolidation pressures: 3 GPa (blue) and 5 GPa (red). It is notable that as the content of iron goes up, the specific area of fcc increases as well. This is explained by the fact that copper is compressed by large amounts of iron and undergoes stronger deformations, which translates into an increased specific surface area of copper. In structures with significant content of copper (and small content of iron), the fcc phase is more abundant and has a negative curvature. In structures with small content of copper (and significant content of iron), fcc phases are less abundant and the fcc phase is in the form of convex clusters with a positive surface curvature.

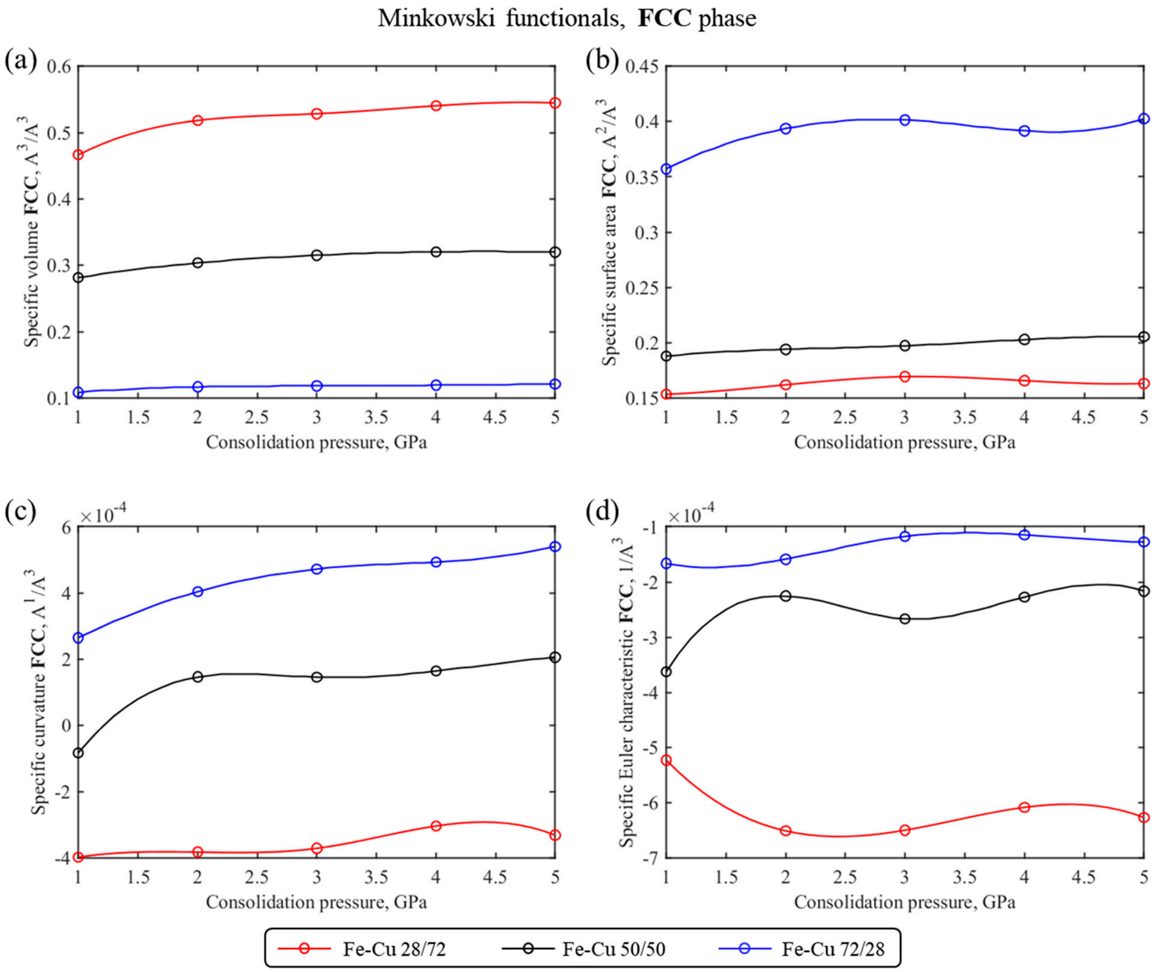

Figure S7. Minkowski functionals for the fcc phase as a function of consolidation pressure: (**a**) M_0(FCC), (**b**) M_1(FCC), (**c**) M_2(FCC), (**d**) M_3(FCC). The results are presented for three different compositions of consolidated Fe-Cu samples: 28/72 (red), 50/50 (black) and 72/28 (blue).

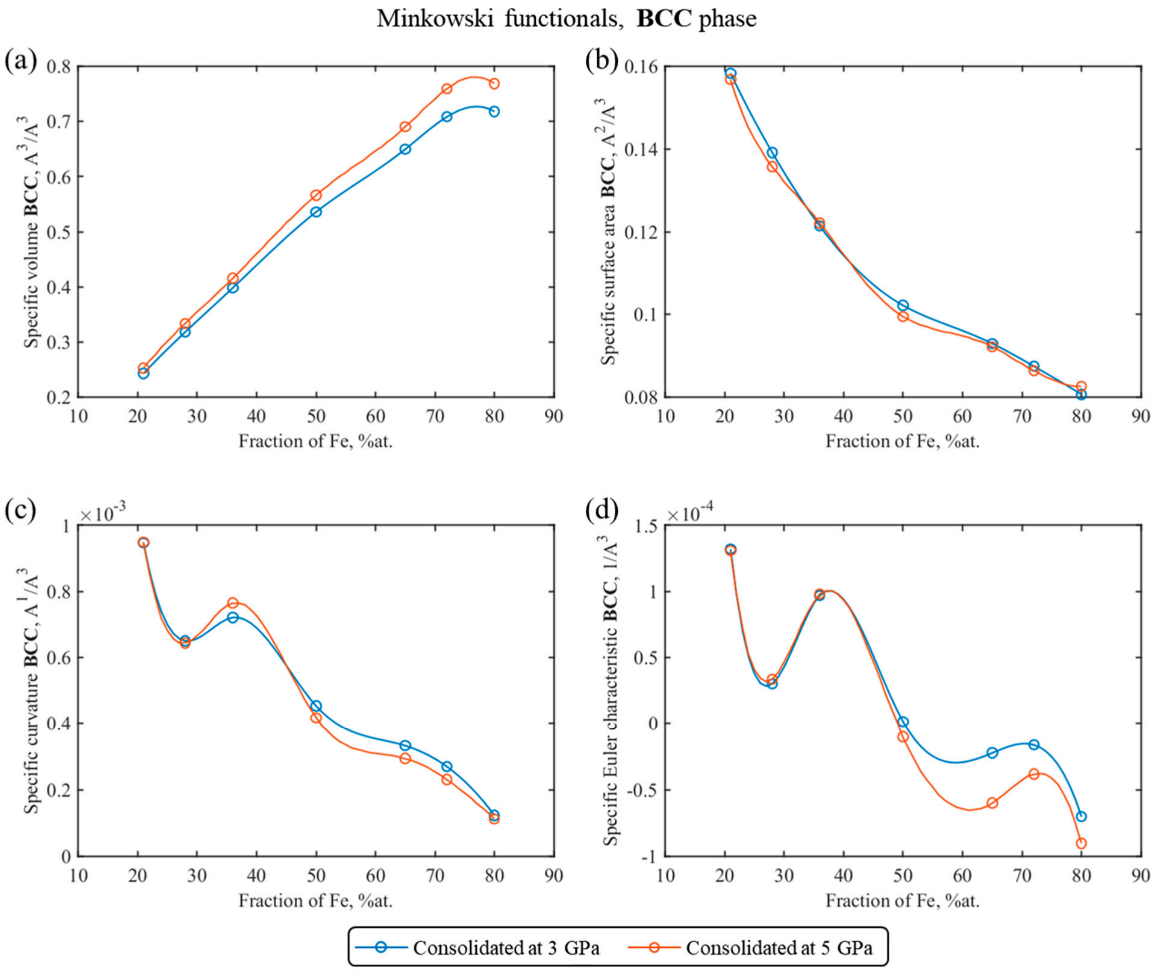

Figure S8. Minkowski functionals for the bcc phase as a function of iron content in consolidated samples: (**a**) M_0(BCC), (**b**) M_1(BCC), (**c**) M_2(BCC), (**d**) M_3(BCC). The results are presented for two consolidation pressures: 3 GPa (blue) and 5 GPa (red). The behavior of the topological characteristics of the bcc phase is close to the behavior of the characteristics of Fe. As the content of iron in the structure is increased, the number of large clusters and grains of iron increases as well, leading to a reduced surface curvature and reduced specific surface area. Also, the sign of the Euler characteristic changes from positive to negative, which can be attributed to increased bondage of the bcc phase because of increased content of Fe in the material.

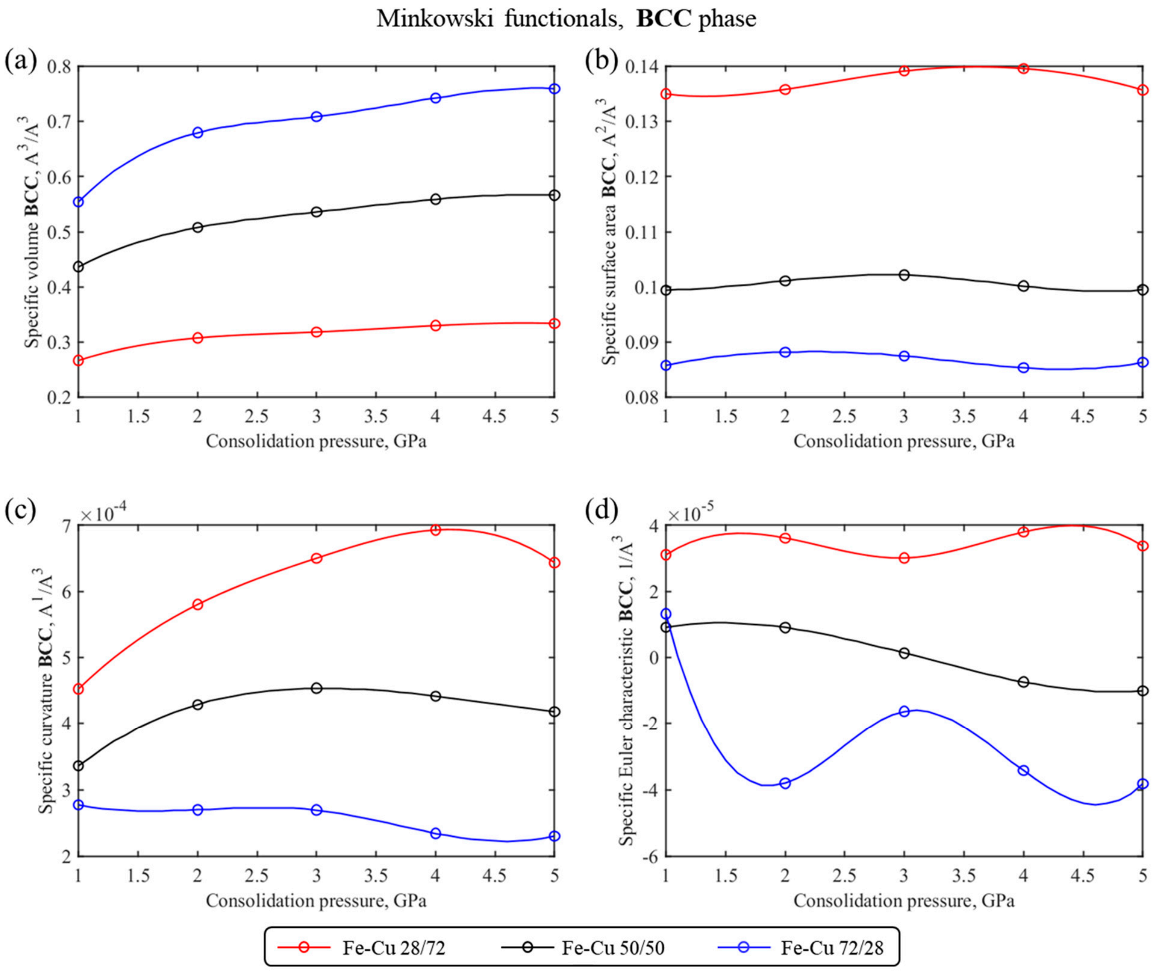

Figure S9. Minkowski functionals for the bcc phase as a function of consolidation pressure: (**a**) M_0(BCC), (**b**) M_1(BCC), (**c**) M_2(BCC), (**d**) M_3(BCC). The results are presented for three different compositions of consolidated Fe-Cu samples: 28/72 (red), 50/50 (black) and 72/28 (blue).

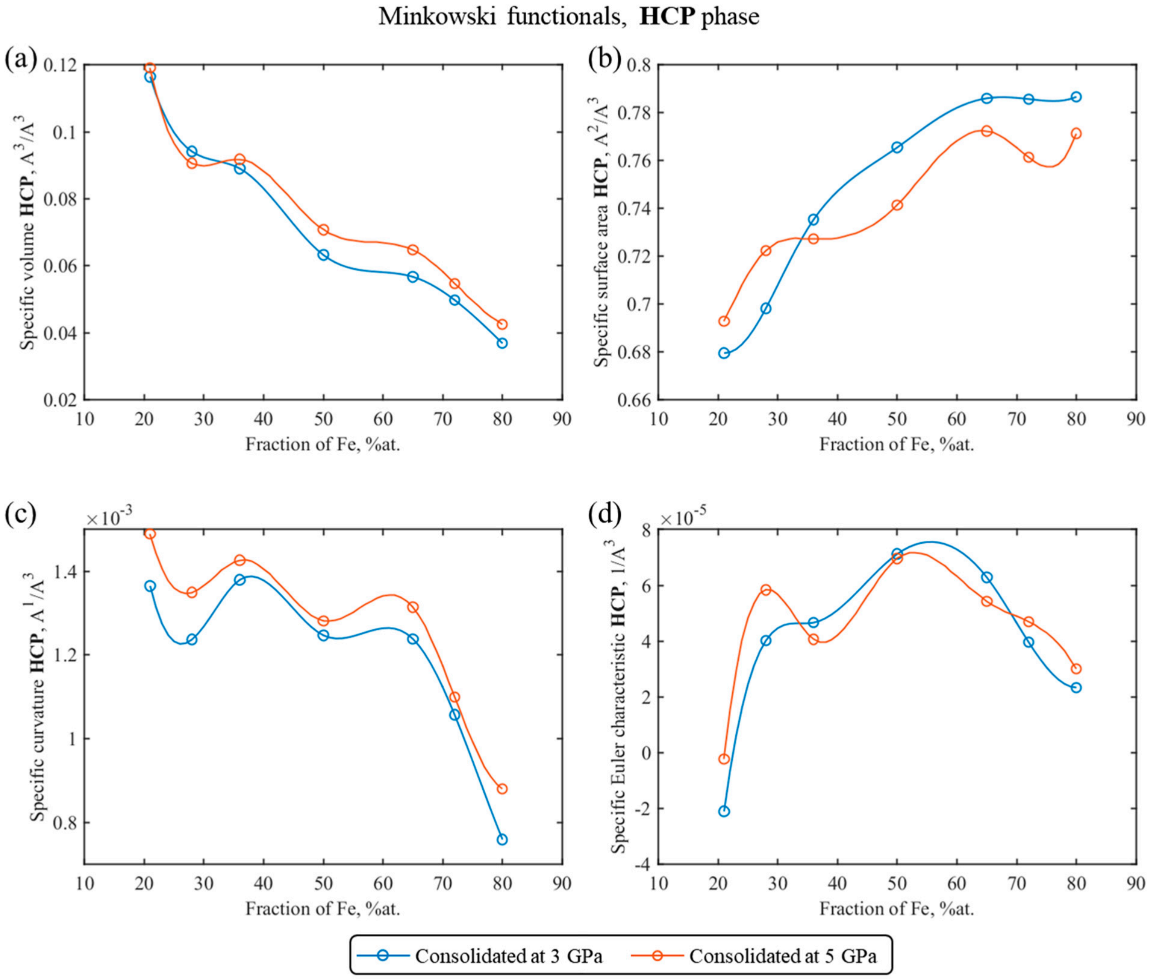

Figure S10. Minkowski functionals for the hpc phase as a function of iron content in consolidated samples: (**a**) M_0(HCP), (**b**) M_1(HCP), (**c**) M_2(HCP), (**d**) M_3(HCP). The results are presented for two cases of consolidation pressures: 3 GPa (blue) and 5 GPa (red). The hcp phase is represented by thin protracted layers inside the copper phase with positive specific curvature as their shape is identical to the cross-sections of areas filled with copper. As the content of iron in the structure increases, the specific volume of hcp decreases since the total volume of copper (inside which fcc-hcp two boundaries emerge) is reduced. The specific area of the hcp surface grows as the content of iron is increased.

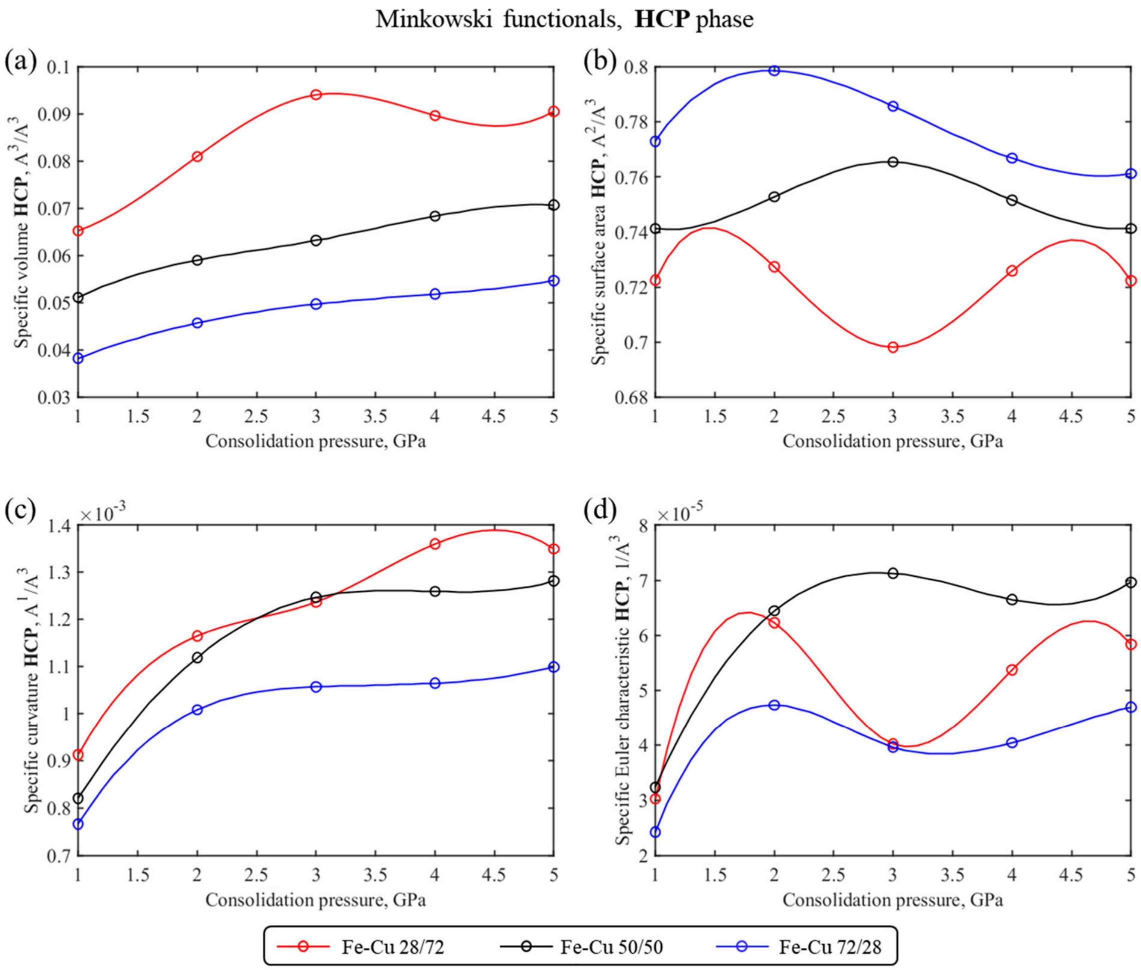

Figure S11. Minkowski functionals for the hcp phase as a function of consolidation pressure: (**a**) M_0(HCP), (**b**) M_1(HCP), (**c**) M_2(HCP), (**d**) M_3(HCP). The results are presented for three different compositions of consolidated Fe-Cu samples: 28/72 (red), 50/50 (black) and 72/28 (blue).

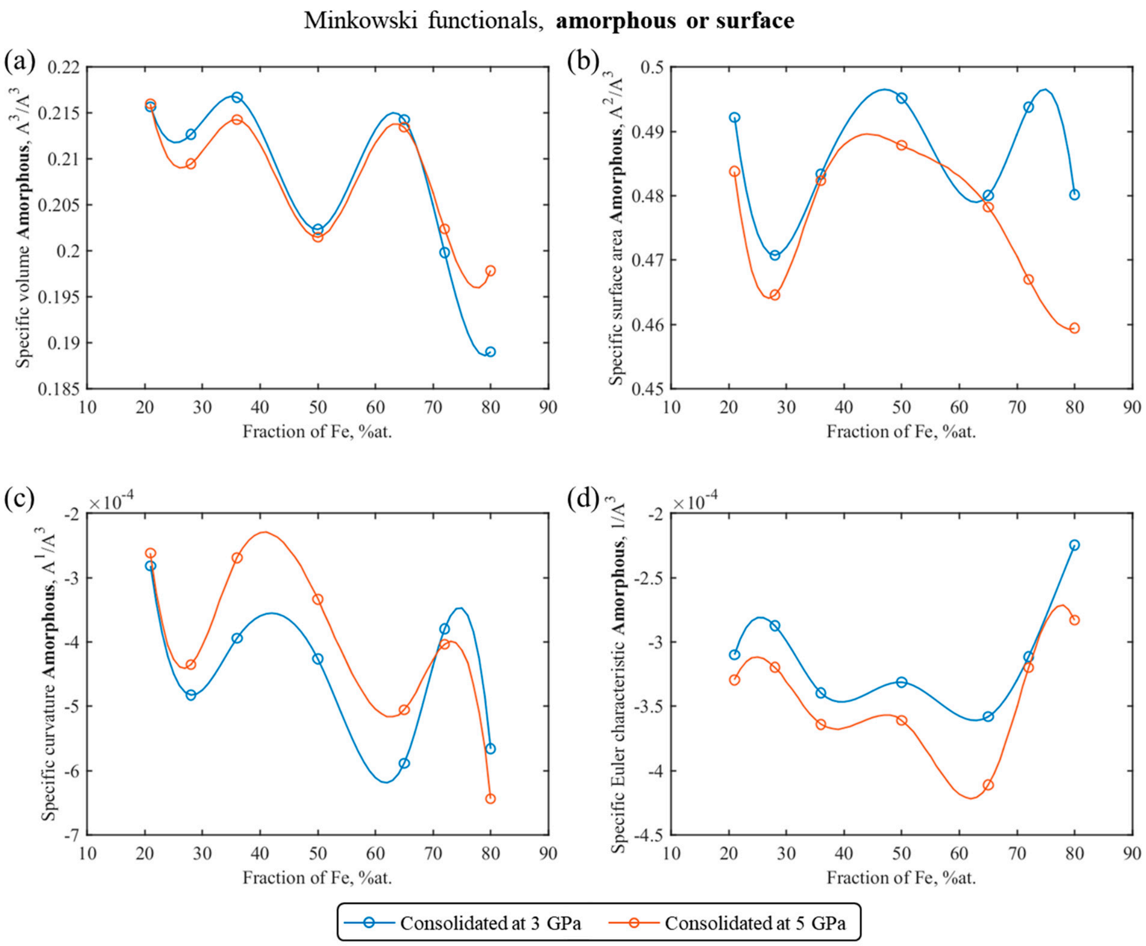

Figure S12. Minkowski functionals for the disordered phase (including the amorphous phase and the surface atoms) as a function of Fe content in the consolidated samples: (**a**) M_0(disordered), (**b**) M_1(disordered), (**c**) M_2(disordered), (**d**) M_3(disordered). The results are presented for two consolidation pressures: 3 GPa (blue) and 5 GPa (red). Negative curvature values of the unordered phase suggest that its surface is concave, and negative values of the Euler characteristic suggest that the unordered phase consists of large protracted clusters.

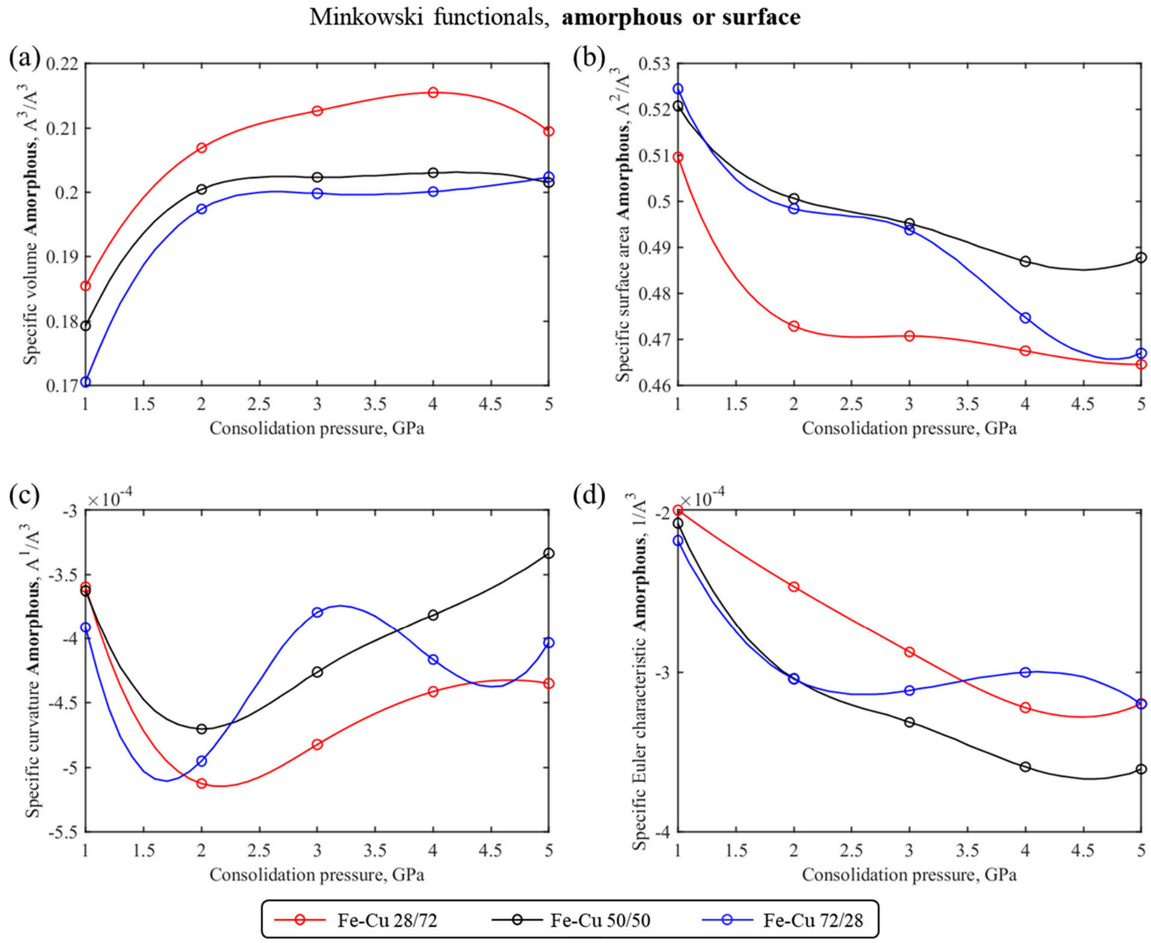

Figure S13. Minkowski functionals for the disordered phase (including the amorphous phase and the surface atoms) as a function of consolidation pressure: (**a**) M_0(disordered), (**b**) M_1(disordered), (**c**) M_2(disordered), (**d**) M_3(disordered). The results are presented for three different compositions of consolidated Fe-Cu 28/72 (red), 50/50 (black) and 72/28 (blue) samples.

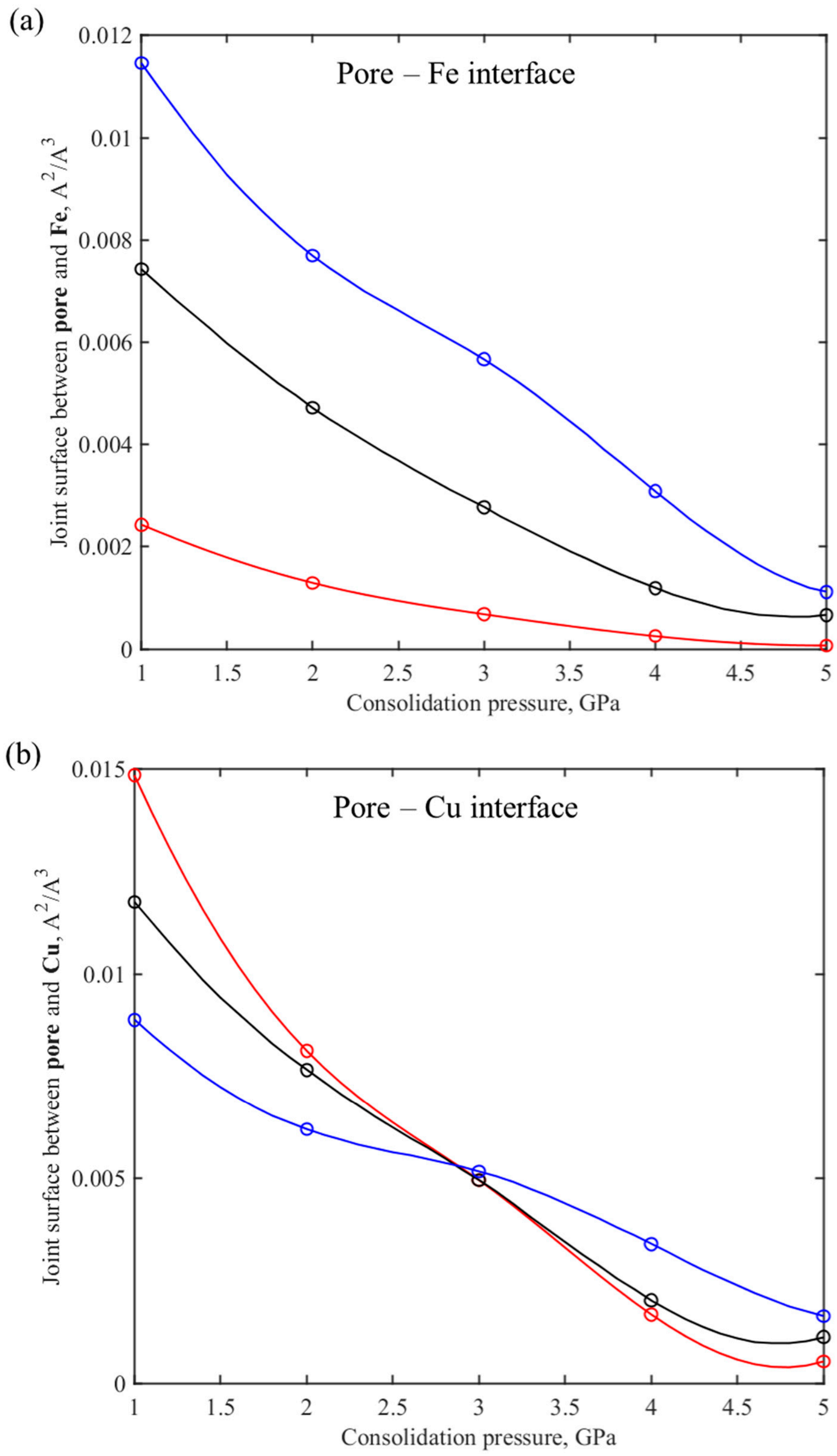

Figure S14. Areas of pairwise pore-iron (**a**) and pore-copper (**b**) interfaces as a function of consolidation pressures for three elemental compositions.

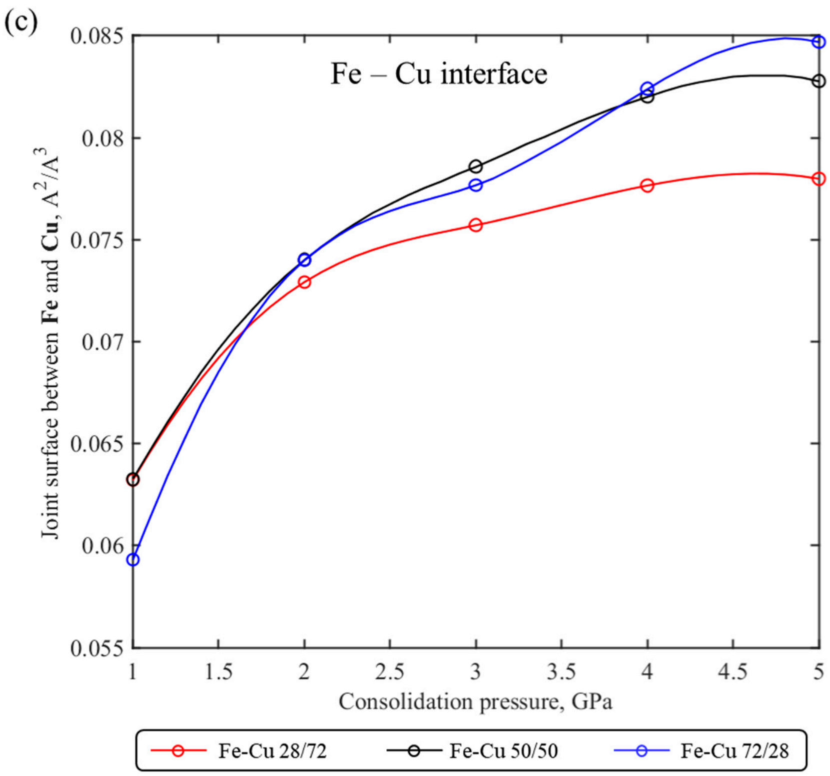

Figure S14. Continuation: (**c**) the area of bi-material (iron-copper) interface as a function of consolidation pressures for three elemental compositions.

6. EXAMPLE: The Use of Minkowski Functionals Library for the *Prediction* of Fe-Cu Nanocomposites Density

It is obvious that the absolute mass density ρ of the bimetallic composite depends on only zero Minkowski functionals M_0^{Fe} and M_0^{Cu}, which are the relative volumes of the components. Despite the simplicity of prediction of the density ρ of Fe-Cu composite, we nevertheless consider it as an example, since in present study the only this physical property (ρ) was numerically estimated together with Minkowski functionals for all 23 model samples (Table S2). Following by the Scheme I, the next step is to solve the system of 8 (or more) linear equations (*), in which $M_i^{Fe}(p_j, r_j)$ and $M_i^{Cu}(p_j, r_j)$ are known (Figures 8, S4, S5), and the right-hand side is the corresponding values of density $\rho(p_j, r_j)$, $j = 1, \ldots, N$ (in eight or more points $N \geq 8$ from Tables S3, S4):

$$\sum_i a_i^{Fe} M_i^{Fe}(p_1, r_1) + \sum_i a_i^{Cu} M_i^{Cu}(p_1, r_1) = \rho(p_1, r_1),$$
$$\ldots \qquad (*)$$
$$\sum_i a_i^{Fe} M_i^{Fe}(p_N, r_N) + \sum_i a_i^{Cu} M_i^{Cu}(p_N, r_N) = \rho(p_N, r_N),$$

where $i = 0, 1, 2, 3$, and N is number of equations (points), p_j – consolidation pressure, r_j – Fe-to-Cu ratio.

Using, for example, 14 values of ρ calculated for 7 samples with different Fe-to-Cu ratio r_j consolidated at 2 different pressures p_j = 3 and 5 GPa (Table S4) and 112 (=14×4×2) values of $M_i^{Fe}(p_j, r_j)$ and $M_i^{Cu}(p_j, r_j)$ from the Minkowski functionals library (which is available at Tsukanov-Lab webpage https://tsukanov-lab.moy.su or via link https://yadi.sk/d/1TdJHckC0XlKNg), we obtain the overdetermined system of $N = 14$ linear equations with 8 unknown variables a_i^{Fe} and a_i^{Cu}, $i = 0$, 1, 2, 3. The solution of such a system is a vector $\boldsymbol{a}$ which minimizes the maximum absolute value of the residuals, $\boldsymbol{a}$ can be found using, for example, an algorithm [Barrodale, I.; Phillips, C. Algorithm

495: solution of an overdetermined system of linear equations in the Chebychev norm [F4]. *ACM Transactions on Mathematical Software* (*TOMS*), **1975**, *1*(*3*), 264-270.].

The result of solving the system (*) is presented in Table S5.

Table S5. The coefficients a of the expansion of the absolute density ρ in series of Minkowski functionals.

	a_0, 10^3 kg/m³	a_1, 10^{-6} kg/m²	a_2, 10^{-12} kg/m	a_3, 10^{-21} kg
Fe	7.957	-49.85	0.900	-1.68
Cu	8.981	-43.50	0.271	-3.90

As it can be seen from the Table S5, the property ρ is mainly determined by zero Minkowski functionals M_0^{Fe}, M_0^{Cu}, since corresponding coefficients a_0 are 9 and more orders of magnitude larger than coefficients $a_{i>0}$, as it was expected. Moreover, the values of a_0^{Fe} and a_0^{Cu} are almost equal to the densities of bulk iron and copper, respectively.

Let consider three cases with simple geometry of the materials, for which the Minkowski functionals can be found analytically: I – pure Fe, II – pure Cu and III – spherical iron inclusion in copper matrix with Fe-to-Cu ratio $r = 1$ (Figure S15a). It is easy to verify that Minkowski functionals for cases I and II are as follows: $M_0^{Fe} = 1$, $M_{i>0}^{Fe} = 0$, $M_i^{Cu} = 0$, and $M_0^{Cu} = 1$, $M_{i>0}^{Cu} = 0$, $M_i^{Fe} = 0$, respectively. Thus, the density *predictions* of pure Fe and Cu samples are $\rho_{Fe}^* = M_0^{Fe} a_0^{Fe}$= 7957 kg/m³ and $\rho_{Cu}^* = M_0^{Cu} a_0^{Cu}$= 8981 kg/m³, respectively.

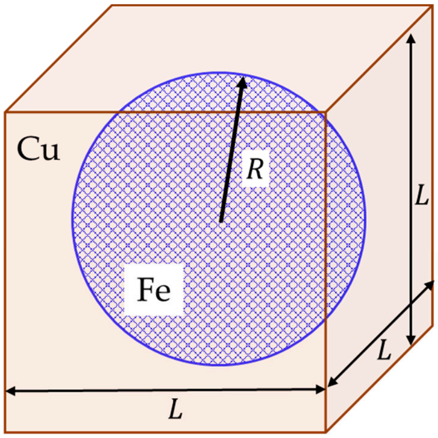

(a)

$$M_0^{Fe} = M_0^{Cu} = \frac{1}{2},$$
$$M_1^{Fe} = M_1^{Cu} = 4\pi R^2$$
$$M_2^{Fe} = -M_2^{Cu} = 2R$$
$$M_3^{Fe} = 1, \quad M_3^{Cu} = 2$$
$$L = 1, \quad R = \frac{1}{2}\sqrt[3]{3/\pi}$$

(b)

Figure S15. Case of Fe-Cu composite with simple geometry: (**a**) periodic cell is spherical Fe inclusion in Cu matrix, (**b**) Minkowski functionals for Fe and Cu phases.

The Minkowski functionals of case III are presented at Figure S15b. Using these values and coefficients from Table S5, it can be found that the *prediction* of ρ for case III composite is 8469 kg/m³, which coincides with high accuracy with the value ${}^1\!/_2\,(a_0^{Fe}+a_0^{Cu}) = {}^1\!/_2\,(\rho_{Fe}^* + \rho_{Cu}^*)$, since the mass density is a property which is independent on internal surfaces, interface curvature and Euler characteristics of the bicomponent material.
